# The potato cyst nematode *Globodera pallida* overcomes major potato resistance through selection on standing variation at a single locus

**DOI:** 10.1111/nph.70886

**Published:** 2026-01-06

**Authors:** Arno S. Schaveling, Dennie M. te Molder, Paul Heeres, Joris J. M. van Steenbrugge, Stefan J. S. van de Ruitenbeek, Casper C. van Schaik, Sven van den Elsen, Geert Smant, Mark G. Sterken

**Affiliations:** ^1^ Laboratory of Nematology, Wageningen University & Research Droevendaalsesteeg 1 6708 PB Wageningen the Netherlands; ^2^ Averis Seeds B.V. Valtherblokken‐Zuid 40 7876 TC Valthermond the Netherlands; ^3^ Department of Genetics University Medical Center, Utrecht University Heidelberglaan 100 3584 CX Utrecht the Netherlands

**Keywords:** effector, *Globodera pallida*, *GpaV*_*vrn*_, potato, resistance, standing variation, virulence

## Abstract

- *Globodera pallida* poses a major threat to potato production, with management strategies primarily relying on genetic resistance. However, increasing virulence in field populations across Western Europe raises major concerns for *G. pallida* control.
- To investigate the evolutionary mechanisms driving this rise in virulence, we propagated 13 field populations on 30 commercial potato varieties. Our findings indicate that the genetic basis of resistance in potatoes is small, with the major resistance conferred by *GpaV* from *Solanum vernei*. The wide application of *GpaV*_*vrn*_ has led to continuous selection on standing genetic variation in *G. pallida*.
- To map virulence, we propagated two field populations on a *GpaV*_*vrn*_‐resistant variety for five generations. High‐coverage whole‐genome sequencing of each generation revealed that *GpaV*_*vrn*_‐mediated selection acted on a single locus of a newly assembled *G. pallida* Rookmaker reference genome. Examination of this virulence‐associated locus identified *Gp‐pat‐1* as a candidate gene. Silencing *Gp‐pat‐1* increased virulence on a *GpaV*_*vrn*_‐resistant variety but had no effect on nematode virulence on a susceptible variety, classifying *Gp‐pat‐1* as an avirulence gene.
- Our findings show that *GpaV*_*vrn*_‐mediated negative selection on *Gp‐pat‐1* is driving the emergence of virulence and improves our understanding of resistance breakdown and the evolutionary dynamics of nematode adaptation in the field.

## Introduction

The potato cyst nematode (PCN) species *Globodera pallida* and *G. rostochiensis* threaten European potato production (Hockland *et al*., 2012; Jones *et al*., 2013). PCN infestations cause symptoms that include stunted growth, leaf yellowing, and reduced tuber size, leading to substantial yield losses (Price *et al*., 2021). The use of resistant potato varieties has long been the cornerstone of PCN management (Gartner *et al*., 2021). Following a major *G. pallida* outbreak in the 1990s, resistant varieties that carried resistances from wild *Solanum* species were introduced to control *G. pallida* (Grenier *et al*., 2020). However, the deployment of these resistances has exerted strong selection pressure on *G. pallida* populations. Over the last decade, resistance‐breaking *G. pallida* populations have become a matter of great concern (Niere *et al*., 2014; den Nijs & van Heese, 2019; Mwangi *et al*., 2019a). Identifying the genetic basis of potato resistance and the mechanism through which *G. pallida* overcomes that this is critical for PCN adaptation and durable resistance deployment.

Plants and plant pathogens are in a constant evolutionary ‘arms race’, in which plants evolve defence mechanisms and pathogens counter‐evolve strategies to evade or suppress them (Ngou *et al*., 2022). In agricultural settings, resistance (R) genes used for pathogen control are typically introgressed from wild material into commercial cultivars. On the other side, pathogens produce so‐called effector molecules, which aim to evade the plant immune response. When (R) proteins recognise pathogen‐derived effectors, they activate immune responses restricting pathogen growth and minimising tissue damage. In turn, to escape detection and maintain infectivity, pathogens adapt by altering or losing recognised effectors. The deployment of R genes exerts a strong selective pressure on pathogen populations, leading to the emergence of virulence. Such resistance breakdown can arise through *de novo* mutations or selection on standing genetic variation (Brown, 2015).

Potato resistances typically affect PCN feeding site formation. As PCN are obligate sedentary endoparasites, their survival relies on the development of a syncytium. The syncytium is formed through the dissolution of plant cell walls and subsequent fusion of neighbouring protoplasts (Goverse *et al*., 2000). Mature syncytia can consist of hundreds of fused host cells, supplying nutrients critical for nematode development and maturation. Disrupting syncytium formation or functioning is an effective mechanism of resistance (Goverse & Smant, 2014). Resistant potato varieties generally induce strong necrosis around the head of sedentary juveniles to prevent syncytium initiation, or around the syncytium to hinder syncytium development, arresting juvenile development (Rice *et al*., 1987; Fournet *et al*., 2018; Varypatakis *et al*., 2020). Although numerous sources of potato resistance against *G. pallida* have been mapped ((Gartner *et al*., 2024; Leuenberger *et al*., 2025) and reviewed in Gartner *et al*. (2021)), the genetic basis of potato resistance in commercial varieties has yet to be defined.

Resistance‐breaking *G. pallida* field populations have been reported in Emsland, Germany (Niere *et al*., 2014) and north‐eastern Netherlands (den Nijs & van Heese, 2019; Grenier *et al*., 2020). These populations have been reported to break through resistances in varieties that were previously identified as resistant based on standard *G. pallida* testing populations. Techniques to study nematode adaptation include genome scans for signatures of selection (Eoche‐Bosy *et al*., 2017b; Varypatakis *et al*., 2020; Kwon *et al*., 2024b) and comparative transcriptomics (Kwon *et al*., 2024a; Lechevalier *et al*., 2025b). Previously, many studies have explored phenotypic and genetic adaptations of *G. pallida* to resistant potato varieties in experimental settings (Fournet *et al*., 2013, 2018; Eoche‐Bosy *et al*., 2017a, 2017b; Mwangi *et al*., 2019a; Varypatakis *et al*., 2020; Lechevalier *et al*., 2025a, 2025b). However, the genetic basis and molecular mechanisms underlying virulence on resistant potato varieties are still poorly understood.

In this study, we aimed to identify the genetic basis of *G. pallida* resistance in commercial potato varieties and resolve the cause of increasing virulence in Dutch *G. pallida* field populations. We discovered the source of resistance, *GpaV*_*vrn*_, is shared by all resistant potato varieties tested. We tested the hypothesis that *G. pallida* virulence is caused by selection on standing variation already present at the time of introduction of the resistance varieties. We associated virulence with a single locus on the newly assembled *G. pallida* reference genome. To identify putative resistance‐breaking effector genes within the avirulence locus, we applied three criteria: (1) their likeliness of being secreted, (2) their expression during early infection, and (3) the presence of allelic variation. This pinpointed *Gp‐pat‐1* as a candidate gene for avirulence on *GpaV*_*vrn*_. Silencing *Gp‐pat‐1* in juveniles significantly increased virulence on resistant potatoes, confirming its role in the breakdown of *GpaV*_*vrn*_.

## Materials and Methods

### *Globodera pallida* populations

Thirteen populations of *Globodera pallida* (Stone) were collected from infection foci in fields with PCN resistant potato varieties. These populations were gathered from 2011 to 2015 and originate from the northeast of the Netherlands. All populations were propagated on the susceptible potato variety Desirée and confirmed to consist exclusively of *G. pallida* through species‐specific PCR testing. The Dutch population Pa_3_‐E400 (Rookmaker) and the European population Pa_3_‐Chavornay, which are commonly used for selecting PCN resistant breeding material, were included for reference.

The E400 Rookmaker population was also used for construction of a new reference genome. The current best reference genome is based on the Pa_2_‐D383 population (Van Steenbrugge *et al*., 2023). However, this population is genetically distant from most *G. pallida* populations currently and historically identified (Folkertsma *et al*., 1996).

### Potato varieties

Thirty‐one different resistant and widely grown commercial potato varieties were assessed across four standard PCN resistance tests for quantifying reproduction of PCN on potato (EPPO, 2021). The presence of the *GpaV*_*vrn*_ resistance within these potato varieties (except the susceptible reference variety Desiree) was confirmed with a genetic marker by breeding companies.

### General notes on data analysis

All tests and analyses were conducted in ‘R’ (v.4.1.0 win x64) using Rstudio (v.1.4.1717; R Core Team, 2013; Team, 2015). Only for the alignment of the sequencing data and variant calling, other software was used. In R, the tidyverse packages, especially ggplot2 and dplyr, were used for data processing in general and generation of most of the figures. All scripts are available through gitlab (https://git.wur.nl/published_papers/Schaveling_2025_Pallifit_virulence).

### Standard PCN resistance tests

Four standard PCN resistance tests were conducted according to the EPPO standard protocol PM 3/68 (2), (EPPO, 2021). The resistance tests are ordered based on appearance in the results section. For all four experiments, single potato eye plugs were planted into 2‐l pots containing *c*. 2000 g of soil inoculated with five juveniles per gram of soil. The pots with the potato plants were maintained in a glasshouse under natural light conditions from February to May. One hundred days after the start of the experiment, the soil was allowed to dry slowly. The cysts were extracted from the dry soil by elutriation, whereafter the number of cysts per pot and the number of living juveniles and eggs per cyst were quantified. The number of living larvae and eggs was counted in triplicate.

The first standard PCN resistance tests were executed in 2019 at the Stichting Nederlandse Algemene Keuringsdienst voor Zaaizaad en Pootgoed van Landbouwgewassen (NAK) (Emmeloord, the Netherlands). The goal of this PCN resistance test was to compare the multiplication of two previously tested virulent *G. pallida* populations (i.e. AMPOP02 and AMPOP13) and three newly collected field populations (i.e. 2017.Pa.2018, 2017.Te.2018, and 2017.dC.2018) with two standard *G. pallida* populations (Pa_3_‐E400 and Pa_3_‐Chavornay) on 16 commercial potato varieties (i.e. Alcander, Allison, Altus, Ardeche, Arsenal, Avarna, Avito, Axion, Basin Russet, Desiree, Festien, Innovator, Libero, Lugano, Seresta, and Supporter). The PCN resistance test included four replicates for each combination of potato variety and nematode population.

The second standard PCN resistance test was executed in 2017 at the Hilbrands Laboratorium B.V. (HLB) (Wijster, the Netherlands). Herein, the multiplication of nine field populations of *G. pallida* (i.e. AMPOP01, AMPOP02, AMPOP03, AMPOP06, AMPOP09, AMPOP10, AMPOP13, AMPOP15, and AMPOP18) was tested on 15 commercial potato varieties with strong *G*. pallida resistance according to the national variety list of the Netherlands (i.e. Altus, Ardeche, Arsenal, Avarna, Avito, Axion, Basin Russet, Festien, HZD 06–1249, Innovator, Libero, Seresta, Supporter, and VD 07‐0289) and the reference variety Desiree. Each combination of potato variety and *G. pallida* population was replicated three times.

The year after, in 2018, a third PCN resistance test was conducted at the HLB with 7 *G. pallida* populations (i.e. AMPOP01, AMPOP02, AMPOP03, AMPOP06, AMPOP09, AMPOP10, and AMPOP18) and 16 commercial potato varieties (i.e. Actaro, Avatar, Aveka, Aventra, BMC, Desiree, Festien, Merenco, Novano, Saprodi, Sarion, Sereno, Seresta, Simphony, Stratos, and Vermont) using the same protocol.

The fourth PCN resistance test was conducted at the NAK in 2019. The goal of the fourth PCN resistance test was to quantify an increase in multiplication rate of five consecutive generations of two *G. pallida* field populations selected on Seresta (i.e. AMPOP02 and AMPOP10) on a set of six potato varieties (i.e. Avarna, Desiree, Festien, Innovator, Libero, and Seresta). For this experiment, each combination of potato variety and *G. pallida* population was replicated three times. We corrected four errors in the quantification of the AMPOP10 propagation (AMPOP10DS_2016). The dilutions made for counting were incorrect. This led to calculating extreme amounts of nematodes per cyst (> 500) in the Desiree, Seresta, Festien, and Avarna pots. However, the cyst counting data were not affected; therefore, we could reconstruct the dilution mistakes (one twofold and three fourfold mistakes) by relying on the propagation in AMPOP10D and AMPOP10DS2 (as the within‐population number of cysts per larvae per variety were stable).

### Data normalisation of standardised PCN resistance tests

To make integrated analysis possible, the second and third standard PCN resistance tests were normalised based on the three potato varieties that were measured on seven identical *G. pallida* field isolates in each year. For each year, the average Pf/Pi, number of cysts, and number of larvae were calculated over these potato varieties and *G. pallida* populations and the ratio over the years was calculated to provide a normalisation constant by:

$$N_{\text{year}}=\bar{\frac{P_{\text{year},i,j}}{\bar{P_{\text{total},i,j}}}}$$

where *N*_year_ is the normalisation constant, and $\bar{P_{\text{year}}}$ is the average phenotype over potato varieties *i* (Desiree, Festien, and Seresta) and *G. pallida* populations *j* (AMPOP01, AMPOP02, AMPOP03, AMPOP06, AMPOP09, AMPOP10, and AMPOP18). $\bar{P_{\text{total},i,j}}$ is the total average over the 2 years. The phenotypes were normalised by:

$$P_{\mathrm{norm},i,j}=\frac{\bar{P_{i,j}}}{N_{\text{year}}}$$

where $\bar{P_{i,j}}$ is the trait average over three replicates of potato variety *i* (one of the remaining 28 potato varieties), and *G. pallida* population *j* (one of 10 populations).

### Data analysis of standardised PCN resistance test

Differences between groups were determined using two‐sided *t*‐tests as implemented in the R‐package ggpubr (Kassambara, 2018).

A cluster analysis was conducted to compare the potato varieties and *G. pallida* populations. A Euclidian distance matrix was calculated based on the averaged normalised Pf/Pi values per potato variety per *G. pallida* population; using the *dist* function in ‘R’. These were clustered using the *hclust* function in ‘R’. To further test the clusters, a pairwise Tukey test was conducted, for which the *P*‐values were corrected for multiple testing using Benjamini–Hochberg correction as implemented in the *p.adjust* function in ‘R’. For plotting, the *P*‐values were log_10_‐transformed, the minimum untransformed *P*‐value was capped at *P* = 1*10^−10^.

Analyses of variance were done using a PERMANOVA, for which we used the *adonis*2 function of the vegan package (Oksanen *et al*., 2013). The PERMANOVA model for analysis of the contributions factors to virulence was run on the normalised propagation data (Pf/Pi) using

$$P_{\frac{\mathrm{Pf}}{\mathrm{Pi}},i,j}=\begin{array}{l} {\mathrm{Cl}}_{i}+S_{\mathrm{SER},j}+V_{i}+{\mathrm{Gp}}_{j}+{\mathrm{Cl}}_{i}\times S_{\mathrm{SER},j}+{\mathrm{Cl}}_{i}\\ \times \;{\mathrm{Gp}}_{j}+S_{\mathrm{SER},j}\times V_{i}+V_{i}\times {\mathrm{Gp}}_{j} \end{array}$$

where *P*_Pf/Pi_ is the normalised propagation of *G. pallida* population j (one of nine virulent populations) on potato variety i (one of 28 potato varieties). Cl is the cluster the potato variety belongs to (Cl_SER_, CL_FES_), S_SER_ is the mean Pf/Pi on Seresta‐cluster potato varieties. *V* is the potato variety (one of 28), Gp is the *G. pallida* population (one of nine). The significances were calculated using 10 000 permutations. It should be noted that this model excluded the two varieties that were not in the Seresta and Festien cluster (Desiree and Aventra). As these two varieties were very susceptible, their cluster captures a high amount of variance. Hence, inclusion led to a higher amount of variance captured by cluster.

### Potato pedigree analysis and SNP data

Potato pedigrees were obtained via the potato pedigree database (Van Berloo *et al*., 2007). The pedigree contains information on 28/31 potato varieties tested in the presented experiments (Supporting Information Table S1; HZD 06–1249, Novano, and VD 07‐0289 were not included in the database and excluded from the analysis). The whole database was downloaded and initially processed using custom scripts to map the resistance progenitors of the potato varieties. The progenitors were manually annotated for underlying wild potato sources, focusing on sources with known resistance contributions: *Solanum multidissectum*, *S. oplocense*, *S. sparsipilum*, *S. spegazzinii*, *S. tarijense, S. tuberosum* ssp. *andigena*, and *S. vernei* (van der Voort *et al*., 1998; Rouppe van der Voort *et al*., 2000; Gartner *et al*., 2021). Subsequently, these sources were mapped back to current varieties.

For 22 of the 31 potato varieties tested in the presented experiments, additional single‐nucleotide polymorphism (SNP) data were generated by the breeding companies Averis, Agrico, and HZPC. The SNP data were based on the SolSTW SNP array platform (Vos *et al*., 2015), and the potato PGSC v.4.03 pseudomolecules (Xu *et al*., 2011; Sharma *et al*., 2013). The data were analysed by graphical mapping, where based on known phenotypes areas with shared breakpoints for introgression are identified. This approach is described in van Eck *et al*. (2017). We used the susceptible variety Desiree as a negative control and the resistant variety Innovator as a positive control for *GpaV*_*vrn*_. For *Gpa6*, we used Desiree as a negative control and Festien as a positive control.

### Small container PCN resistance tests

As a service for potato producers, small container tests for PCN resistance in potato are commercially offered at the HLB to predict the performance of potato varieties on infection foci of PCNs. These small container tests for PCN resistance in potato are conducted in 55‐ml transparent plastic containers harbouring a small tuber and soil inoculated with 300 juveniles of *G. pallida* per container. A test includes eight replicates for each combination of potato variety and *G. pallida* population. The containers are kept at 20°C in the dark for 8–12 wk, after which the number of cysts visible on the root system through the walls of the container is counted. A database of anonymised results of all small container PCN resistance tests commercially executed from 2016 to 2021 by HLB was made available to this study. Notably, multiplication rates of nine of the *G. pallida* populations mentioned above (i.e. AMPOP01, AMPOP02, AMPOP03, AMPOP06, AMPOP09, AMPOP10, AMPOP13, AMPOP15, and AMPOP18) on 13 potato varieties (i.e. Avito, Supporter, Festien, Altus, Merenco, Saprodi, BMC, Avarna, Sarion, Seresta, Axion, Novano, and Desiree) in small container tests were also in the database. More importantly, similar data of 125 infestation foci of *G. pallida*, which were recently sampled on farms and tested on the potato varieties Desiree, Seresta, and Festien in small container tests, were retrieved from the database for further analyses.

### Data analysis of the small container tests

For the correlation analysis of the small container tests vs the standardised container tests, the mean relative susceptibility (RS) values (determined vs Desiree) were calculated for either test. Thus, there were RS values for both tests. Subsequently, we calculated the correlation of the RS values from both tests per *G. pallida* population for the nine virulent populations. A Pearson correlation was calculated as well as the significance of the correlation. We also calculated the correlation when excluding Desiree (as, per definition it has an RS = 100). Therefore, the correlation excluding Desiree is a better estimate of how well virulence levels correlate.

The between variety differences (Desiree, Seresta, and Festien) were calculated using a *t*‐test on the RS values.

### Promethion sequencing and assembly of *G. pallida* Rookmaker genome

DNA isolation of the *G. pallida* Rookmaker population was performed as described previously (Steenbrugge *et al*., 2023). Long‐read DNA sequencing was done by USEQ using Oxford Nanopore promethION (the Netherlands). Raw reads (ERR15205749) were corrected to merge haplotypes using the correction mode in Canu (Koren *et al*., 2017) by reducing the error rate to a maximum of 14.4% and the corrected coverage to a minimum of 400.

Genome assembly and polishing were conducted as previously described (Steenbrugge *et al*., 2023). In brief, using wtdgb2 v.2.5 (Ruan & Li, 2020), multiple initial genome assemblies were generated based on the corrected Nanopore reads while manually refining the parameters minimal read length, k‐mer size, and minimal read depth. These parameters were optimised to generate an assembly close to the expected genome size of *G. pallida* (110 Mb). After optimisation, for *G. pallida*, a minimum read length cut‐off of 4000, minimal read depth of 12, and a HPC k‐mer size of 19 was used. Remaining haplotigs were pruned from the assemblies using Purge Haplotigs v.1.1.2 (Roach *et al*., 2018). Based on the histogram (Fig. S1), the low read‐depth cut‐off parameter was set at 75, the low point between haploid and diploid peak was determined at 375, and the read‐depth high cut‐off was set at 650. Contigs were improved using FinisherSC v.2.1 (Lam *et al*., 2015) at default settings and scaffolded using LongStitch v.1.0.4. with a HPC k‐mer size of 32 and a window size of 500 (Coombe *et al*., 2021).

The scaffolded genome was filled with RagTag v.2.1.0 (Alonge *et al*., 2022) using the closely related *G. pallida* D383 genome (Steenbrugge *et al*., 2023); the resulting assembly was polished with Nanopore reads by Medaka v.1.4.4 (https://github.com/nanoporetech/medaka), model r941_min_high_g360, followed by five iterations of polishing with Pilon v.1.24 (Walker *et al*., 2014) using Illumina HiSeq reads from the Rookmaker population. Gene annotations were predicted using Braker v.2.1.6 (Brůna *et al*., 2021) with a modified intron support of 0.51, aided by RNA‐seq data of parasitic and preparasitic Rookmaker life stages. The data used included the following: Rookmaker samples of E‐MTAB‐11646 (Zheng *et al*., 2022), life‐stage samples of PRJEB2896 (Cotton *et al*., 2014), and our own generated RNA‐seq of mixed stages of Rookmaker (ERR15277786). We also used the protein data base of OrthoDB (v.10; Kriventseva *et al*., 2018). We used OmicsBox (v.3.0; Götz *et al*., 2008) to annotate protein sequences with Gene Ontology (GO) terms. The genome is deposited at the European Nucleotide Archive (ENA) under PRJEB91928.

### Synteny analysis between the D383 and Rookmaker virulence loci

The synteny between the D383 and Rookmaker genomes was computed using ntSynt v.1.0.2 (Coombe *et al*., 2024) with the expected percentage divergence (*−d*) set at 3. Synteny was visualised using ggplot2 (v.3.5.2, https://git.wur.nl/molde006/nemasynt). For whole‐genome synteny, only syntenic blocks larger than 300 kb of D383 scaffolds 1–19 were used for visualisation. For synteny of the virulence locus, only syntenic loci of D383 scaffold 2 and Rookmaker scaffold 28 larger than 10 kb were used for visualisation.

### Selection for virulence by *GpaV*_*vrn*_

Two *G. pallida* populations collected from infection foci in fields with PCN resistant potato varieties, designated AMPOP02 and AMPOP10, were used to further select for virulence on the *GpaV*_*vrn*_‐containing potato variety Seresta in pot experiments. To this end, a maximum of 10 000 eggs was inoculated into 2‐l pots containing a 2.2 kg mixture of silver sand, kaolin, hydro grains and nutrients. To each of the pots, a potato piece containing a single shoot was added. To obtain sufficient starting material, before the selection experiment, AMPOP02 was propagated on the susceptible variety Desiree for two consecutive generations, resulting in AMpop02D_2_. AMPOP10 was propagated on Desiree for one generation, resulting in AMpop10D_1_.

The selection experiment started in 2015, going through one generation per year, leading to fourth‐generation selection populations at the end of 2018. For each generation, we aimed to save cysts to later use for DNA extraction. Since all of the yield from the first round of selection on Seresta (S_1_) was needed for inoculation of the second generation, we could not save cysts from these populations. To be able to include an S_1_ population in further analyses, we again inoculated the unselected starting populations on Seresta to reproduce the S_1_. However, it should be noted that the S_1_ populations were independently derived starting from the same Desiree propagation.

### Phenotypic data analysis of the *GpaV*_*vrn*_ ‐selection experiment

The data of the fourth standard PCN resistance test were analysed for three phenotypes that could be relevant for virulence: the propagation (Pf/Pi), the RS vs Desiree (RS in percentage), and the number of eggs/juveniles per cyst. We had material to test consecutive selection generation S_2_–S_4_ for both AMPOP02 and AMPOP10. We did test a nonselected and a S_1_ population, as these were not directly related to generation S_2_–S_4_.

Within variety comparisons were conducted using Kruskal–Wallis tests. To test for the contribution of generations of selection a PERMANOVA was conducted on Generations 1, 2, 3, and 4 for AMPOP02 and AMPOP10 separately using the model:

$$\frac{\mathrm{Pf}}{\mathrm{Pi}},i,j={\mathrm{Cl}}_{i}+C_{i}+{\mathrm{Gs}}_{j}$$

where Pf/Pi is the propagation of *G. pallida* population j (either AMPOP02 or AMPOP10) on potato variety i (one of 6 potato varieties), Cl is the cluster the potato variety belongs to, *C* is the potato variety, Gs is the generation of Seresta selection of the *G. pallida* population (1–4). The significances were calculated using 10 000 permutations.

### Broad‐sense heritability analysis

Broad‐sense heritability was calculated for the potato varieties Desiree, Seresta, Festien, Avarna, Libero, and Innovator for the fourth standard PCN resistance test on field populations, the selection experiment, and the small container tests (only Desiree, Seresta, and Festien). The heritability was determined independently for the AMPOP02, and the AMPOP10 selected populations. We used the equation

$$H^{2}=\left(\frac{V_{g}}{V_{g}+V_{e}}\right)$$

where *H* ^2^ is the (broad sense) heritability, *V*_g_ is the variance explained by population, and *V*_e_ is the residual variance. The *V*_g_ and *V*_e_ components were determined using a REML model as implemented by lme4: RS ~ 1 + (1|population; Bates *et al*., 2015). It should be noted that depending on the populations used, the interpretation of *H* ^2^ varies. Namely, in the standardised PCN resistance test on various populations as well as the small container tests, it is a measure of variance captured by populations for which we did not include their genetic relations. It was therefore a less accurate estimation of the true heritability. The *H* ^2^ for the selection experiments was the most reliable estimator of the heritability, as the populations were directly related.

### DNA isolation and sequencing of *GpaV*_*vrn*_ ‐selected *G. pallida* populations

DNA of the *G. pallida* selection experiment was isolated using previously published protocols (Steenbrugge *et al*., 2023). In short, over 10 000 preparasitic second stage juveniles (ppJ2s) collected after counting were frozen and stored at −80°C. These were lysed (Holterman *et al*., 2006) and DNA was isolated using phenol/chloroform/isoamyl alcohol. DNA quantities were determined using Qubit Fluorometer (Invitrogen). Because we were dependent on how well the material was preserved after counting, not all samples provided DNA of sufficient quantity and/or quality; of the 60 samples collected (2 populations * 2 potato varieties * 5 generations * 3 replicates), 45 processed and successfully sequenced.

The DNA was sequenced at BGI Genomics (China) using BGISEQ‐500 with a median output of 295.4 million reads of 100 bases (paired) per sample. Upon arrival, sample integrity and purity were tested via gel‐electrophoresis. Per sample 1 μg of DNA was fragmented by Covaris and fragments in the size ranges of 150–250 bp were selected which were subsequently quantified by Qubit. Fragments were end‐repaired and 3′ adenylated before adaptor‐ligation to the 3′ end. The fragments were amplified by PCR and purified with the Agencourt AMPure XP‐Medium kit. DNA was quantified by Agilent 4200 TapeStation. Double‐stranded PCR products were circularised, and the circular DNA was used as input for sequencing in the BGISEQ‐500 platform. FASTQ files were deposited at the ENA (E‐MTAB‐15408).

### Sequence alignment and variant calling of the *GpaV*_*vrn*_ ‐selection experiment

DNA sequencing reads of the samples from the *GpaV*_*vrn*_‐selection experiment were aligned to the newly assembled Rookmaker genome and the previously constructed *G. pallida* D383 genome (Steenbrugge *et al*., 2023) using bwa‐mem2 (v.2.2.1; Vasimuddin *et al*., 2019) with default parameters. Duplicate reads were marked using Samtools MarkDups (v.1.14; Danecek *et al*., 2021). Variants were called using Bcftools (mpileup & call, v.1.14; Danecek *et al*., 2021) with the default parameters except for a maximum number of 1500 reads per position (‐d), an INDEL threshold of 1500 reads (‐L), and a minimum mapping quality of 20 (‐q) after mismatch correction (‐C50). The initial round of vcf filtering was performed using Bcftools's vcfultils.pl with the default parameters except for a minimum read depth (‐d) of 10 and minimum alt allele depth (−a) of 5. For the remaining calls, the alternative allele fraction (AAF) was computed by dividing the allelic depth (AD) of the most common alternative allele (across all samples) by the combined AD of the reference allele and most common alternative allele (bi‐allelic depth). Single calls were marked as missing if the fraction of bi‐allelic reads was less than 0.95 or if the bi‐allelic depth was less than 5. The resulting variant matrix spanned a continuous range from 0 to 1, which was better able to represent the allele fractions present in heterogeneous populations. The pipeline was orchestrated by Snakemake (v.6.8.0; Mölder *et al*., 2021) running on python (v.3.7.8; https://git.wur.nl/stefan.vanderuitenbeek/dnaseq_variant_calling_snakemake_pipeline).

A second round of filtering was applied to the variant matrix based in part on statistics generated by vcftools (v.0.1.16; Danecek *et al*., 2011), including a minimum quality of 30, a minimum and maximum mean read depth of 30 and 300, respectively, at least 10 alternative reads, SNP variants only (as these are more reliably called), mean alternative allele frequencies between 0.05 and 0.95, and no missing calls. These filtering steps led to the detection of 1216 526 variants on the Rookmaker genome and 1132 495 variants on the D383 genome.

### Analysis of genetic variation of the *GpaV*_*vrn*_ ‐selection experiment

To explore patterns of genetic differentiation among the samples, the Rookmaker variants were analysed in a principal component (PC) analysis using *prcomp* with the parameter scale = TRUE. The first four axes (capturing 96.9% of variance) were graphically analysed.

The generational model applied on both sets of variants was a linear model, where the assumption was that in the range where we were measuring, the allele frequency increased linearly per generation. This assumption would not be true at either very low or very high RS. Since we measured relative susceptibilities within the range of 18.6 till 80.3 on Seresta (Table S2), it is likely that our measurements are in the range where allele frequencies increase in a more‐or‐less linear relation with generation (Schouten, 1993). Therefore, we used:

$$A_{x}=G_{x}+e$$

where *A* was the allele frequency in sample *x* (one of 23 from AMPOP02 and one of 22 from AMPOP10), *G* was the generation of sample *x* (0, 1, 2, 3, 4, or 5), and *e* is the error‐term. The obtained significances were Bonferroni‐corrected. This model was run separately for AMPOP02 and AMPOP10.

The locus containing the virulence gene was determined based on changepoint analysis, using the changepoint package in R (Killick & Eckley, 2014). The association outcomes of AMPOP02 and AMPOP10 were combined. We used the associated variant density to identify the most SNP‐dense associated region on scaffold 28.

The Venn diagram illustrating the overlap in significant variants derived from both population AMPOP02 and AMPOP10 independently was created with the *VennDiagram* package in R.

Linkage between variants was calculated as the squared Pearson correlation coefficient of the allele frequencies per sample of the variant of interest (*i.e*. the three overlapping variants) with allele frequencies per sample of the other variants on scaffold 28. The threshold of *R* ^2^ = 0.8 contained the top 1% correlation coefficients.

### Protein sequence analyses for examining the genes on the avirulence locus

Signal peptide prediction was carried out on the amino acid sequences using SignalP v.6.0 selecting ‘Eukarya’ as organism (Teufel *et al*., 2022). Transmembrane prediction was carried out using TMHMM v.2.0 on default settings (Krogh *et al*., 2001). BLASTx was performed with the coding nucleotide sequences against the nonredundant protein sequences (nr) of the NCBI with default parameters (blast.ncbi.nlm.nih.gov/Blast.cgi). BLASTn was performed with the genomic transcript nucleotide sequences against the nucleotide collection (nr/nt) of the NCBI with default parameters (blast.ncbi.nlm.nih.gov/Blast.cgi). *Gp‐pat‐1* domain predictions and GO term annotations were conducted with Interpro (Paysan‐Lafosse *et al*., 2023).

### Temporal transcriptome analysis in avirulent and virulent *G. pallida* juveniles

To assess the transcriptome of preparasitic and parasitic *G. pallida* juveniles, *in vitro* infection assays were performed as described previously (Zheng *et al*., 2022). In brief, Rookmaker and AMpop02_DS4_ cysts were hatched based on established protocols (Goverse *et al*., 2000). ppJ2s were collected, cleaned by sucrose purification (Jenkins, 1964), and surface‐sterilised in 0.008% (w/v) mercuric chloride. Fourteen‐day‐old stem cuttings of the potato variety Seresta were inoculated with 100 surface‐sterilised Rookmaker or AMPOP02 ppJ2s. After inoculation, plants were kept in the dark at 18°C, and infected root segments were harvested at 1, 3, 6, and 9 d postinoculation (dpi). Each sample consisted of the infected root tissue pulled from 10 plants. Four independent hatchings were used to conduct four time‐separated biological replicates of the experiment. For each batch, a subsample of the inoculum was used to assess transcription in ppJ2s. Total RNA was extracted with the Maxwell 16 LEV‐plant RNA Kit (Promega), according to the manufacturer's instructions. Samples were sent for transcriptome sequencing to BGI Genomics (China). Stranded libraries were sequenced on the DNBseq G400 platform, generating an average of 77.7 million paired‐end 150 base pair reads per sample (Table S3). The reads were mapped against the *G. pallida* Rookmaker genome (v.1.0), using hisat2 (v.2.2.1; Kim *et al*., 2019). After quality control by MultiQC (Ewels *et al*., 2016), all samples were used for further analysis.

BAM files were loaded into SeqMonk (v.1.48.1, https://www.bioinformatics.babraham.ac.uk/projects/seqmonk/). Raw count values were generated using the RNA‐Seq quantitation pipeline in SeqMonk. Differentially expressed genes were identified by DESeq2 at each of the four parasitic timepoints (Love *et al*., 2014). *P*‐values were corrected for multiple testing (false discovery rate (FDR)) and independent filtering was applied. Transcript per kilobase million (TPM) values were extracted from SeqMonk and loaded into R (v.4.3.2; R Core Team, 2013) for data visualisation.

### Silencing of *Gp‐pat‐1* in preparasitic juveniles

To identify unique regions in the coding sequences (CDS), we performed a BLAST with the CDS against the *G. pallida* Rookmaker CDS database (21 026 sequences). The siRNA generator of Eurofins Genomics (https://eurofinsgenomics.eu/en/ecom/tools/sirna‐design/) was used to design siRNA. The siRNAs are 21 nucleotides long and have an UU dinucleotide at the 3′end (Table S4). Single stranded RNA of the sense and antisense strands was ordered at IDT Europe (Belgium). siRNA targeting *eYFP* was included as a negative control.

Approximately 5000 AMPOP02 juveniles were soaked in 167 μM double‐stranded siRNA, being either 83.3 μM of each of the effector‐targeting siRNA or 167 μM of the *eYFP*‐targeting siRNA. After 2 h incubation, nematodes were surface sterilised with mercury chloride for 20 min. Approximately 100 juveniles were inoculated per 14‐d‐old Desiree or Seresta potato cutting. Infection assays were performed as described previously, with the only change that potato cuttings were grown on Gamborg B5 with 10 g l^−1^ sucrose instead of 20 g l^−1^, as this limited the number of avirulent females that were able to develop on resistant potato varieties.

To assess the expression levels of *Gp‐pat‐1* at 3 dpi, infected root tissue of 10–15 plants was pooled into one biological replicate. RNA was extracted as described before. We synthesised cDNA using GoScript™ Reverse Transcriptase (Promega), and performed qPCR using the iQ™ SYBR® Green Supermix (Bio‐Rad) according to the manufacturer's protocols. Primer sequences for housekeeping genes *Gp_AMA1* and *Gp_GR* were obtained from Sabeh *et al*. (2018) (Table S4). *Gp‐pat‐1* expression levels were normalised based on the expression of the two housekeeping genes using the 2^−ΔΔC^_T_ method (Livak & Schmittgen, 2001). We normalised the gene expression per batch based on the median expression measured in the *eYFP*‐silenced plants. The experiment was replicated twice, with each experiment containing three biological replicates of two different treatments (*eYFP* or *Gp‐pat‐1* siRNA) on two potato varieties (Desiree and Seresta).

To assess the effect of *Gp‐pat‐1* silencing, females were counted at 35 dpi. Phenotypic count data were batch‐normalised against the mean count of the *eYFP*‐treated juveniles inoculated on Desiree. Correction factors were scaled across batches to the overall mean of the two batches to enable comparison across experiments.

### Comparison with *Heterodera schachtii*

To assess gland expression of poly‐A polymerases in cyst nematodes, gland cell RNA‐sequencing data of *Heterodera schachtii* was obtained from the ENA (PRJEB71499; Molloy *et al*., 2024). The data were mapped against the *H. schachtii* Bonn reference genome (PRJNA522950; Siddique *et al*., 2022) and processed as described above.

To identify *H. schachtii* genes with homology to *Gp‐pat‐1*, the *Gp‐pat‐1* protein sequence was used as input in a BLASTp against the Bonn genome, on the WormBase ParaSite server (version: WBPS19; Howe *et al*., 2017). To identify poly‐A polymerases, all genes annotated with poly‐A polymerase activity (GO:1990817) were obtained from WormBase Parasite using the BioMart tool.

## Results

### Commercial potato resistance was overcome by virulent *G. pallida* field populations

**Fig. 1:**
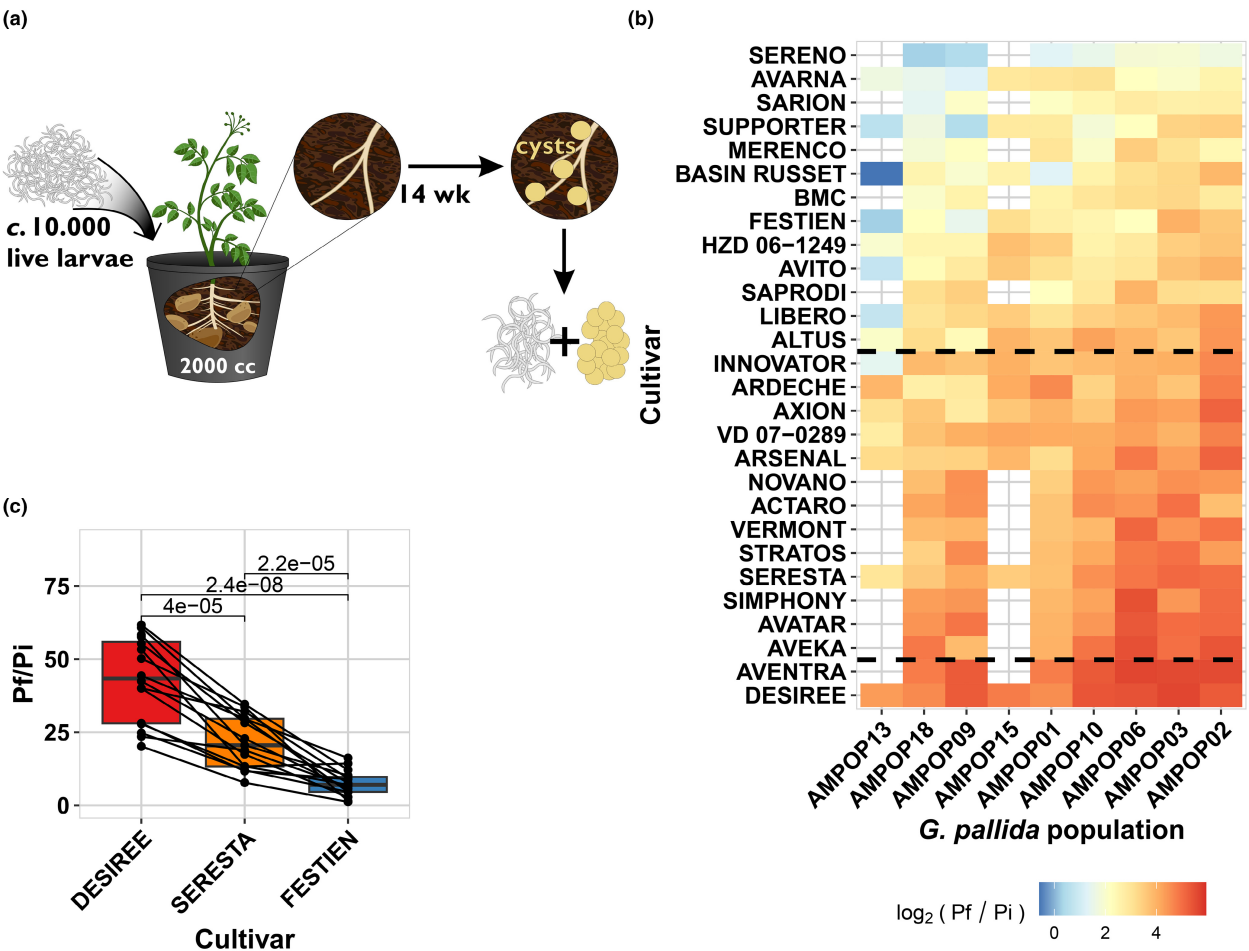
*Globodera pallida* populations from the Netherlands display virulence on resistant potato varieties. (a) The set‐up of the standard potato cyst nematode (PCN) resistance tests. (b) A heatmap of the average log_2_ (Pf/Pi) of the *G. pallida* field populations per potato variety. The blue colours indicate lower propagation, and the red colours indicate higher propagation. The dashed lines indicate the separation between the Desiree‐, Seresta‐, and Festien clusters (Supporting Information Fig. S4). Raw data can be found in Table S6. (c) The propagation (Pf/Pi) of the virulent *G. pallida* field populations on Desiree, Seresta, and Festien. Each dot represents the mean propagation (three replicates) of a *G. pallida* population within a year (2017 or 2018) on the particular variety. The boxplots summarise the data per variety (15–16 datapoints per variety), with each box representing the interquartile range (Q1–Q3; IQR), the horizontal line inside the box marks the median, and the whiskers extend to the smallest and largest nonoutlier values (within 1.5× the IQR from the quartiles). The lines connect the measurements per *G. pallida* population within a year. The significances displayed are from a *t*‐test.

Over the past decade, several European countries have reported the emergence of resistance‐breaking *G. pallida* populations, both in the field and under experimental conditions (Phillips & Blok, 2008; Fournet *et al*., 2013; Niere *et al*., 2014; den Nijs & van Heese, 2019). To assess virulence levels of *G. pallida* field populations on *G. pallida*‐resistant potato varieties, we conducted a first standard PCN resistance test with seven *G. pallida* populations on fifteen Pa2/3‐resistant potato varieties and the susceptible variety Desiree (Fig. 1a; Table S5). The test panel included two standard populations (Chavornay and E400 ‘Rookmaker’) and five field populations. Three *G. pallida* field populations isolated from infection foci in fields with PCN resistant potato varieties (AMPOP02, AMPOP13, and 2017dC) consistently showed higher reproduction rates (P_f_/P_i_) compared with the standard populations (two‐sided *t*‐test, *P* < 0.001; Fig. S2). Since virulence can be defined by a population's reproductive ability on a resistant host relative to its reproductive ability on a susceptible host (Schouten & Beniers, 1997), we concluded that these field populations are virulent on a broad range of potato varieties.

We hypothesise that variations in virulence levels can be explained by differences in the genetic backgrounds of the tested potato varieties. To test this, we conducted a second and third standard PCN resistance test in which we tested nine virulent *G. pallida* field populations on a total of 28 potato varieties. Our results showed that the most virulent *G. pallida* populations were able to propagate on all resistant potato varieties (Figs 1b, S3; Table S6). Moreover, virulence on one resistant variety seems to correlate with virulence on other resistant varieties. To assess these correlations, we performed cluster analysis, including all 28 potato varieties. This revealed three distinct resistance groups (Fig. S4). The groups could be defined by three commonly used varieties for virulence testing: Desiree (no resistance), Seresta (medium resistance), and Festien (strong resistance). Therefore, we named the clusters after these three varieties, where we always saw that the resistance of Festien (Cl_FES_) > Seresta (Cl_SER_) > Desiree (Cl_DES_; Fig. 1c; two‐sided *t*‐test, *P* < 0.001).

Based on these findings, we pursued two lines of further enquiry. First, we investigated the origin of the resistance overcome by virulent *G. pallida* populations. Given our observations, we hypothesised that virulence in *G. pallida* field populations arose from the breakdown of a single resistance shared by all tested potato varieties. Second, we set out to identify the avirulence locus under selection in *G. pallida*. Given the parallel observation of virulent populations in Germany and the Netherlands (Mwangi *et al*., 2019a), we hypothesised that virulence is the result of selection on standing genetic variation, rather than the emergence of novel mutations.

### Virulence in *G. pallida* field populations is based on the breakdown of a single resistance; *GpaV*_*vrn*_

**Fig. 2:**
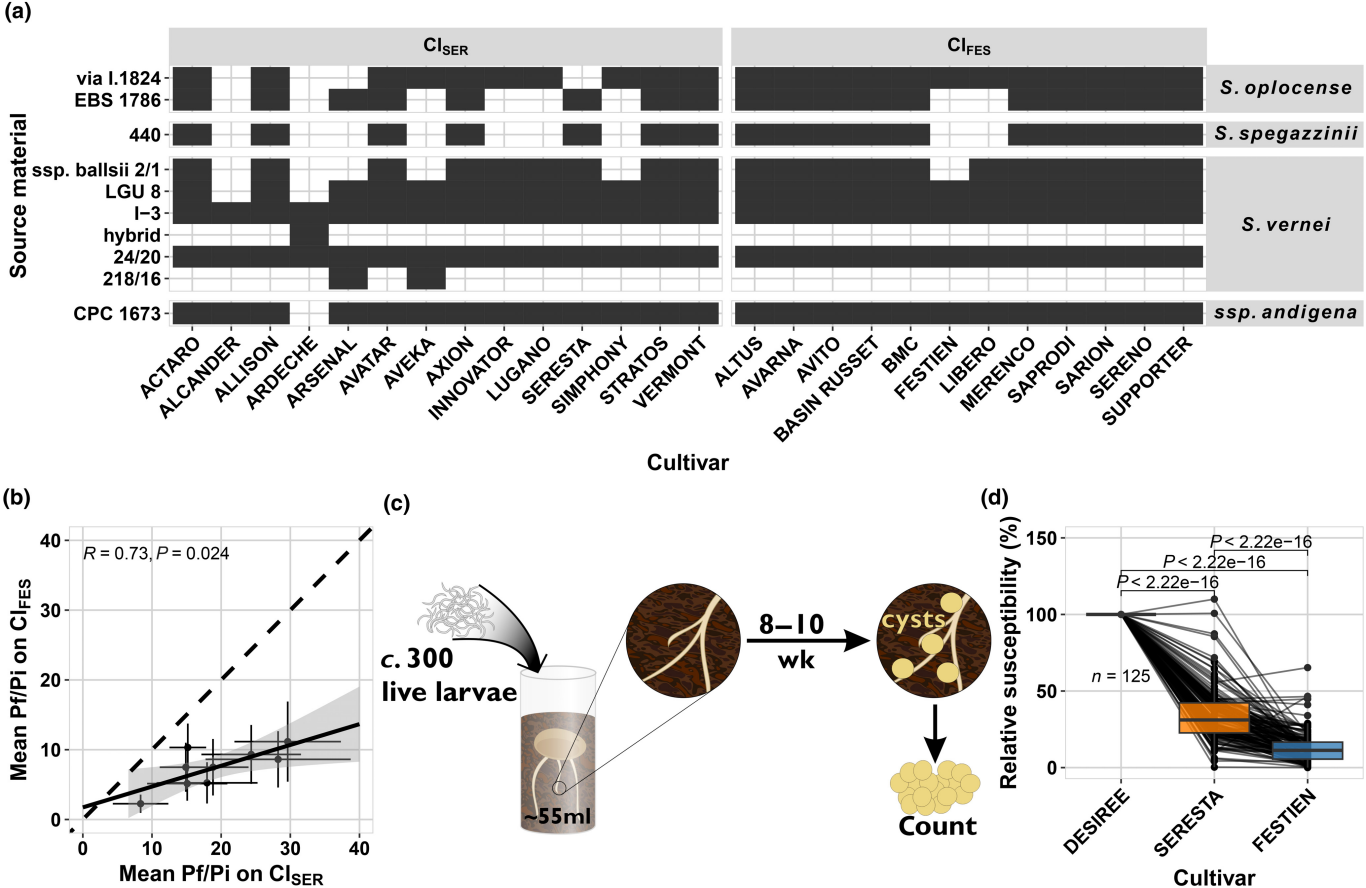
Selection on GpaVvrn can explain virulence in Dutch *Globodera pallida* field populations. (a) The known resistance sources used as progenitors in the tested resistant potato varieties. The data were obtained from the potato pedigree database, which contained pedigrees for 27 of the 31 tested potato varieties (Van Berloo *et al*., 2007). The potato varieties are separated into the previously identified clusters and the progenitors on the species of origin (ssp. *andigena* is a sub‐species of *Solanum tuberosum*). (b) A correlation of the mean propagation (Pf/Pi) of nine *G. pallida* populations on the ClSER varieties with the ClFES varieties. The coefficient of correlation shown is from a Pearson correlation, visualised in the solid black line. The dashed black line is added as a visual aid. (c) The set‐up of the small container tests conducted by HLB B.V. to test potentially virulent field populations of *G. pallida*. (d) The outcome of the small container tests for 125 *G. pallida* populations tested over 2016–2021. Each dot represents the mean relative susceptibility based on 2–16 replicates (median of eight replicates). There were only nine populations where either RSSeresta > RSDesiree or RSFestien > RSSeresta. Each box represents the interquartile range (Q1–Q3; IQR), the horizontal line inside the box marks the median, and the whiskers extend to the smallest and largest nonoutlier values (within 1.5× the IQR from the quartiles). The significances displayed are from a two‐sided *t*‐test.

To determine whether virulence in *G. pallida* field populations arises from the breakdown of a single resistance shared by all potato varieties, we examined the genetic background of the tested potato varieties. Pedigree analysis revealed that all resistant varieties had contributions from various *S. vernei* lines, including LGU 8, 20/24 and I‐3 (Fig. 2a; Van Berloo *et al*., 2007). This finding aligns with previous research that showed that the *GpaV* resistance from *S. vernei* (*GpaV*_*vrn*_) was no longer effective against two French *G. pallida* populations selected for virulence on the *GpaV*_*vrn*_‐resistant potato variety Iledher (Fournet *et al*., 2013). Likewise, *G. pallida* populations recently isolated in Germany displayed virulence on the *GpaV*_*vrn*_‐containing Seresta, similar to the populations described here (Mwangi *et al*., 2019a). We confirmed that all resistant varieties contain *GpaV*_*vrn*_, indicating that *GpaV*_*vrn*_ is a major source of *G. pallida* resistance.

To further verify the presence of *GpaV*_*vrn*_, we deployed graphical genetic mapping to identify genomic introgressions that might contain the R‐gene (van Eck *et al*., 2017). We selected positively for SNPs present in the *GpaV*_*vrn*_‐containing variety Innovator and negatively against SNPs present in the susceptible variety Desiree, after which 1733 SNPs remained, of which 170 were on Chromosome 5. We confirmed that all the resistant varieties contain alternative alleles *c*. 4.7–5.7 Mb on Chromosome 5 of the ST4.03 genome, where *GpaV*_*vrn*_ was previously mapped (Table S7; van Eck *et al*., 2017). As *Gpa6* might also be present, we blasted the CT220 EST (Rouppe van der Voort *et al*., 2000) to the ST4.03 genome, which identified a single region from 60 925 291 to 60 925 562 Mb on Chromosome 9. Given the pedigree, we selected positively for Festien and negatively against Desiree, after which 1755 SNPs remained, of which 154 were on Chromosome 9. There were some shared SNPs *c*. 59.1–61.1 Mb, but it could not be confirmed which varieties carry *Gpa6* and which do not (Table S8). Taken together, this indicates that resistance is based on *S. vernei*, with *GpaV*_*vrn*_ as the most parsimonious explanation for resistance.

If virulence is the result of selection on different resistances, we would expect virulence on one resistance to be independent of virulence on other resistances. Conversely, if selection against a singular resistance drives virulence, then differences in virulence among nematode populations would be expected to reflect a gradient of selection, representing the increase of a single virulence allele. To test this, we correlated the mean virulence measured within Cl_SER_ with the mean virulence measured in Cl_FES_ across *G. pallida* field populations. The virulence levels between the two clusters were strongly correlated (test of correlation, *P* = 0.024; Fig. 2b), indicating that virulence on Cl_SER_ varieties and Cl_FES_ varieties involves the same trait. This confirms that the same resistance, *GpaV*_*vrn*_, has been overcome in all these varieties.

### *Globodera pallida* virulence is the result of selection on standing variation

We hypothesised that virulence arose through selection on standing variation, defined as genetic variation present in *G. pallida* field populations before the introduction of commercial *GpaV*_*vrn*_‐resistant varieties on the market 30 years ago. This implies that virulence alleles share the same origin and have spread across different fields over large distances. Alternatively, virulence may have arisen from a *de novo* mutation after the introduction of *GpaV*_*vrn*_‐resistant varieties, in which case virulence would be restricted to a limited number of populations confined to a relatively small geographic area. To test these hypotheses, we assayed a total of 125 *G. pallida* field populations, collected between 2016 and 2021 using small container tests (Fig. 2c; Table S9). We found that virulence was widespread and followed a similar pattern to that identified by testing nine *G. pallida* populations on 28 potato varieties (Figs 2d, S5). This indicates that a scenario where selection on standing variation took place is the most likely.

Selection on standing variation also implies that virulence levels in a *G. pallida* population correspond to its progression through the *GpaV*_*vrn*_‐selection process, with each generation leading to increased virulence. We tested this first by cluster analysis on the 9 *G. pallida* populations tested on 28 potato varieties (Fig. 1b). This analysis did not yield distinct groups; it instead arranged populations along a gradient from least to most virulent (Fig. S6). This indicates that virulence is a quantitative trait shared among many field populations. Next, we tested how much of the variance could be explained by the following: (1) the averaged virulence of the *G. pallida* population within the Cl_SER_ (Fig. 2b) and (2) the assignment of the potato variety to the two clusters (Fig. S7). These two factors, along with their interactions, accounted for 45.2% of variance in the data (PERMANOVA, *P* < 0.0001; Table S10), leaving only 1.3% of the variance over populations unexplained (PERMANOVA, *P* < 0.0001; Table S10). This suggests that differences between *G. pallida* field populations primarily reflect quantitative variation in their stage of selection of virulence. Collectively, these findings indicate that virulence in *G. pallida* populations is shaped by selection acting on standing genetic variation, present before the introduction of *GpaV*_*vrn*_‐resistant seed potatoes.

### *Globodera pallida* virulence on Seresta is a selectable, heritable trait conferring virulence on a range of potato varieties

**Fig. 3:**
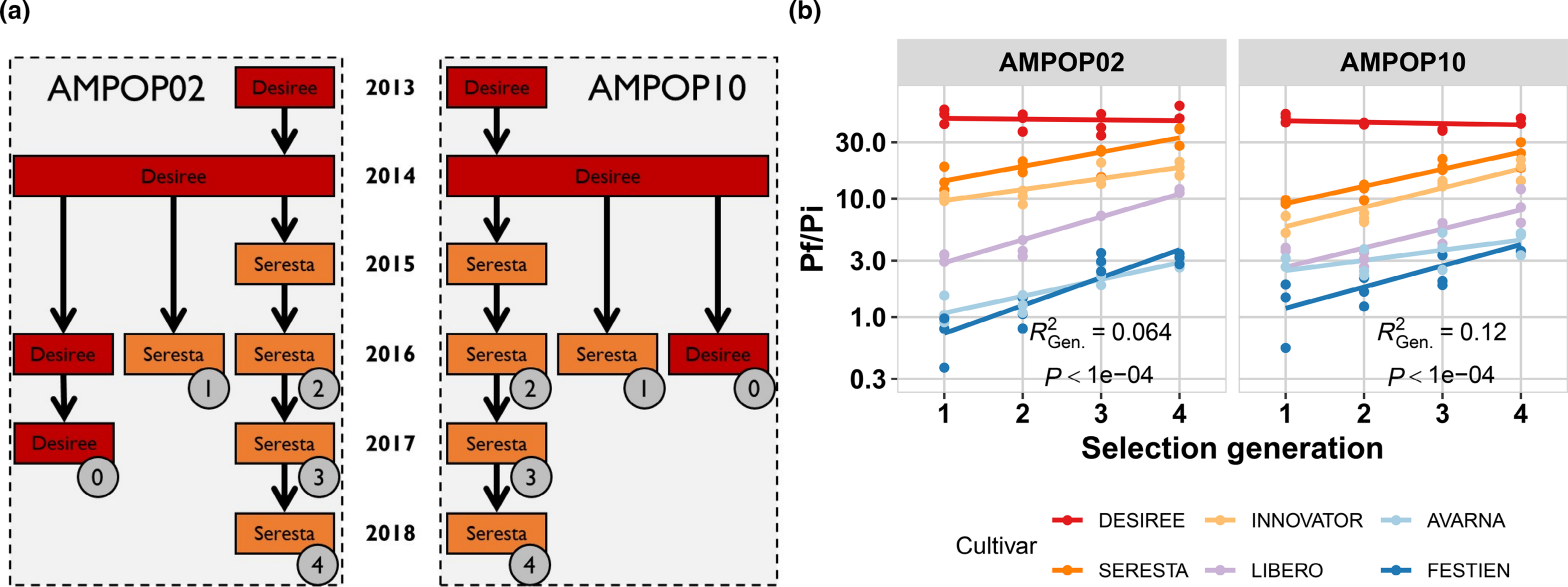
Selection on the *GpaVvrn*‐containing potato variety Seresta also increases virulence on other commercial potato varieties. (a) The selection experiment that was carried out with four generations of selection on Seresta. The colours indicate the potato variety used (Red Desiree, Orange Seresta), and the circles with the numbers indicate which propagation was used in the standard potato cyst nematode (PCN) resistance test. The numbers indicate the generation of selection. Due to the amount of material required for the standard PCN resistance test, Generation 0 and Generation 1 could not be derived from the selection line Generations 2–4 were on. (b) The outcome of the standard PCN resistance test for selected Generations 1–4. Each dot represents the reproduction (Pf/Pi) measured in one 2‐l pot inoculated with 10 000 larvae. The lines are linear regressions added as a visual aid. Colours indicate varieties, where Seresta and Innovator belong to Cl_SER_ and Libero, Avarna and Festien belong to Cl_FES_. The *R* ^2^ is the amount of variance explained by generation as derived from a PERMANOVA. The data can be found in Supporting Information Table S2.

To verify that populations can be positively selected for virulence and to proceed with mapping of the virulence allele, two field populations (AMPOP02 and AMPOP10) that had shown virulence on *GpaV*_*vrn*_ before (Fig. 1d) were further selected on Seresta (which contains *GpaV*_*vrn*_ as major resistance locus (Milczarek *et al*., 2011)). The selection was conducted over the course of 5 yr and yielded populations selected on Seresta for four consecutive generations. Subsets of these populations (where we had sufficient cysts) were tested for virulence in a fourth standard PCN resistance test. For each population, we had three consecutive generations that we could directly compare to determine the increase in virulence (Fig. 3a). Unfortunately, we could not directly compare the unrelated previous generations, as these had been propagated on the nonresistant Desiree variety.

Selection on Seresta increased the virulence of AMPOP02 and AMPOP10 on both Cl_SER_ and Cl_FES_ varieties (Fig. 3b; Table S2). We found that consecutive generations of selection increased the propagation (Pf/Pi) and thereby the RS in multiple potato varieties (Kruskal–Wallis test on Pf/Pi: *P* < 0.05 for 9 out of 10 tests; on RS: *P* < 0.05 for 9/10 tests; Fig. S7a,b). The number of eggs per cyst remained stable over the course of selection (Kruskal–Wallis test on Pf/Pi: *P* > 0.05 for 11/12 tests; Fig. S7c). Consistent with our previous experiments, selection on Seresta resulted in increased virulence, also on the more resistant Cl_FES_ varieties (PERMANOVA, *P* < 0.001; Fig. 3b).

To assess the genetic underpinnings of virulence, we determined the broad‐sense heritability (*H* ^2^) for each potato variety used in the fourth standard PCN resistance tests, the small container tests, and the selection experiment. All analyses indicated a major genetic component to virulence (Table S11). In the selected populations, the *H* ^2^ was 0.73 and 0.83 for RS on Seresta for AMPOP02 and AMPOP10, respectively (Permutation, *q* < 0.01). Given that broad‐sense heritability is typically high in the case of a single allele, this suggests the selection of a single major virulence allele that explains the heritable variation. This allele, when selected for on Seresta, also increased virulence in other potato varieties, indicating the reliance on a shared source of resistance (*GpaV*_*vrn*_) as the genetic basis for *G. pallida* resistance in resistant potato varieties.

### Virulence against *GpaV*_*vrn*_ results from selection at a single locus on the *G. pallida* genome

**Fig. 4:**
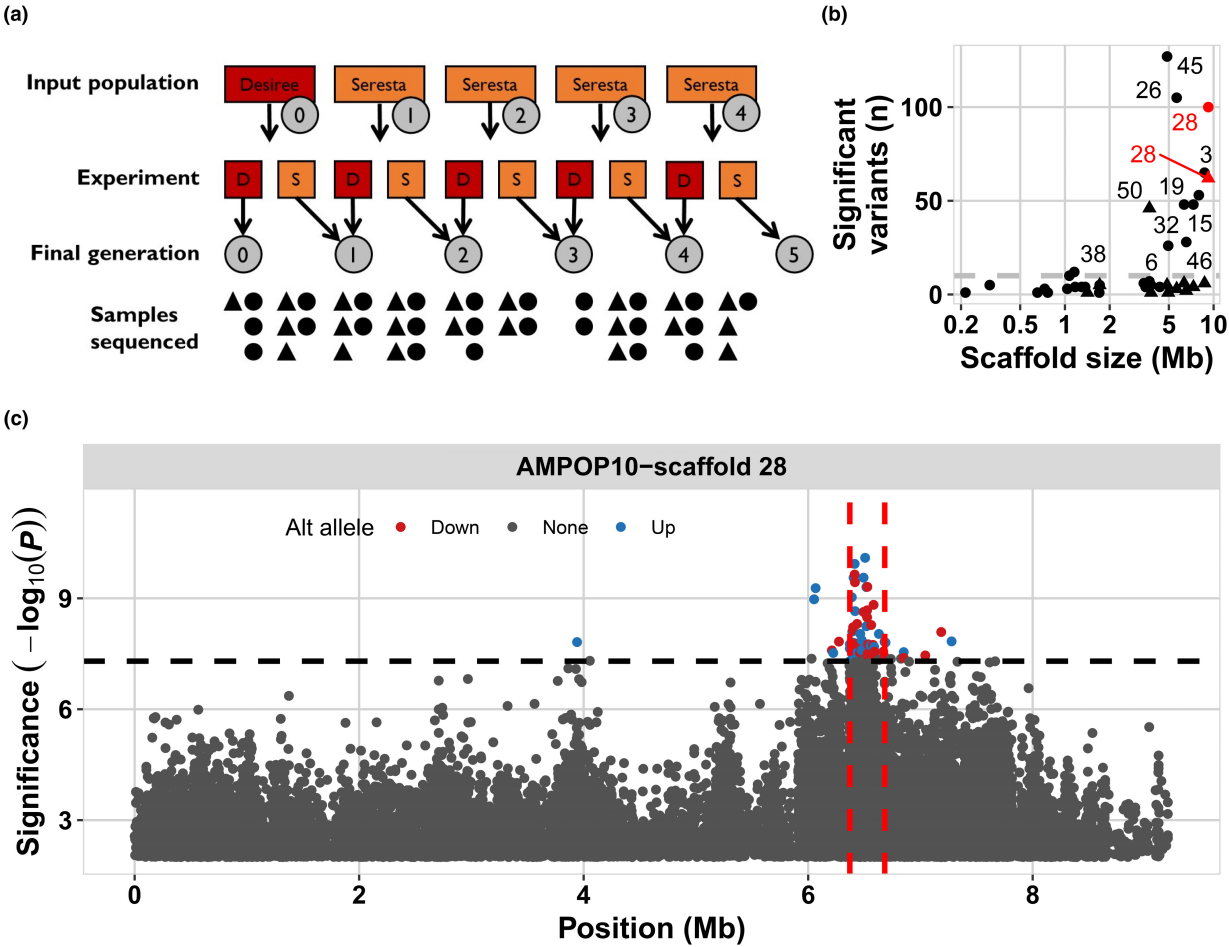
Single genetic locus is selected after repeated selection on Seresta. (a) Overview of the AMPOP02 (dots) and AMPOP10 (triangles) samples generated in the selection experiment and how many were successfully sequenced. As the larvae from which we isolated DNA underwent either a selection on Desiree (red) or Seresta (orange), we took the final number of generations selected on Seresta along in the analysis. (b) The number of variants that significantly correlate with virulence per scaffold plotted against the scaffold size in million bases (Mb). Dots indicate the variants found in AMPOP02, and triangles indicate variants found in AMPOP10. Text indicates which scaffold of the Rookmaker genome the datapoint belongs to. (c) The significance of the variants on scaffold 28 associated with generation in AMPOP10. The variants are plotted on their location on scaffold 28 (in Mb) vs the significance in −log_10_(*P*). The variants where the frequency of the alternative allele is decreasing over generations are coloured red, while those where the alternative allele is increasing are coloured blue. Grey variants are not significant. The dashed horizontal line indicates the Bonferroni‐corrected threshold. The dashed vertical lines indicate the candidate locus. Note that the y‐axis has been cut off at −log_10_(*P*) < 2.

To test whether virulence is the result of selection on a single major virulence allele, we used the selection experiment to detect alternative alleles that were consistently selected for over the generations. To this end, we sequenced *G. pallida* populations collected from the selection experiment. We aimed to sequence all three biological replicates on both Desiree and Seresta separately. This resulted in sequence data for 45 population‐variety combinations (Fig. 4a; Table S12). We used whole‐genome high‐coverage sequencing (achieving a median coverage of 162‐fold per position per sample) to be able to estimate allele frequencies within our populations.

The best currently available *G. pallida* reference genomes is based on the D383 genome (Steenbrugge *et al*., 2023). However, the D383 population is genetically distinct from most other Dutch populations (Folkertsma *et al*., 1996). Therefore, we assembled a new *G. pallida* reference genome that is based on the Dutch Rookmaker (E400) population. The Rookmaker genome is less fragmented and has a higher quality annotation than the previously published D383 genome (Table S13).

Variants were called using the new Rookmaker genome, resulting in the identification of 1017 636 segregating variants within the two selection populations. We used allele frequencies rather than a standard bi‐allelic matrix to be able to explicitly deal with heterozygous populations – not individuals. We used the allele frequencies in a PC analysis to understand the major differences between the 45 samples. We found that the major explanatory component, PC 1 (88.8% of variance) yielded no clear separation and probably captures genetic diversity (heterozygosity) present in both populations (Fig. S8a). PC 2 (8.1% of variance); however, clearly separated the two populations (AMPOP02 and AMPOP10), probably related to alleles distinct for the two populations. Together PC 3 (0.49% of variance) and PC 4 (0.24% of variance), respectively, separated the early generations in AMPOP02 and AMPOP10 from the selected populations (Fig. S8b). This clearly indicates that there was a genetic signal associated with selection.

To identify alleles associated with virulence, we calculated the correlation between alternative allele frequencies and the corresponding generations. For variants associated with virulence, the alternative allele frequencies should increase over the generations of selection as the reference genomes were derived from avirulent *G. pallida* populations. Initially, we identified 680 selected variants in AMPOP02 and 142 selected variants in AMPOP10 (Bonferroni‐corrected *P* < 0.05; Table S14; Fig. S8c). In both populations, scaffold 28 contained many allelic variants that significantly correlated with virulence (Figs 4b, S9). When we investigated the overlap between the significant variants in AMPOP02 and AMPOP10, we found that two variants overlapped, and both were located on scaffold 28 of the Rookmaker genome (hypergeometric test, *P* < 1*10^−4^; Fig. S8d–e). Because these two allelic variants were located outside the CDS of any predicted gene, they were unlikely to be causal for virulence, but may instead be genetically linked to causal variants.

Because of uncertainty in what is continuous sequence, based on synteny between the D383 genome and the Rookmaker genome (Fig. S10), we also conducted the association analysis using the *G. pallida* D383 reference genome (Steenbrugge *et al*., 2023). Here, the majority of the significantly selected variants were found on a region on scaffold 2 of the D383 genome (Fig. S11a–e). We compared the D383 scaffold 2 with the Rookmaker scaffold 28 by assessing the synteny between the two scaffolds (Fig. S11f). The identified regions were syntenic, which gives confidence that we identified a single locus linked to *G. pallida* virulence on *GpaV*_*vrn*_.

We set out to identify the genomic region most likely to contain the virulence allele(s). We analysed the combined association in AMPOP02 and AMPOP10 using changepoint analysis. AMPOP10 contained the narrowest introgression of the virulent haplotype (Fig. 4c), and together with AMPOP02, we found 162 variants on scaffold 28 (Fig. S12a). Using these 162 variants, we found the most variant‐dense locus between 6.37 and 6.68 Mb (Fig. S12b). Indeed, when we analysed the linkage based on the two overlapping SNPs, multiple variants on scaffold 28 showed considerable linkage (*R* ^2^ > 0.8; Fig. S12c,d). The two overlapping variants, on the contrary, were not linked (*R* ^2^ = 0.35), which was expected as one was positively and the other negatively correlated with virulence (Fig. S8d–e). Based on these association analyses, we conclude that the search for the causal gene should prioritise the 311 Kb window on scaffold 28 of the *G. pallida* Rookmaker genome.

### Identification of putative resistance‐breaking effectors on the virulence‐associated locus yields one prime candidate

To identify a virulence allele responsible for breaking resistance, we examined the virulence‐associated genomic locus of *G. pallida* for candidate genes. Automated genome annotation predicted 53 transcripts distributed over 48 genes within the 311‐Kb locus. Due to the inherent limitations of automated genome annotations, the locus was manually inspected and curated following the approach described by (Moya *et al*., 2023), the details of which can be found in Note S1. This resulted in a total of 76 transcripts on 47 genes within the locus (Table S15).

We examined the locus for putative resistance‐breaking genes based on three criteria: (1) their likeliness of being secreted, (2) their expression during early infection, and (3) the presence of allelic variation. These criteria align with the understanding that nematodes secrete effector proteins to avoid or suppress plant defence responses during early infection, and that virulence results from selection on standing variation.

**Fig. 5:**
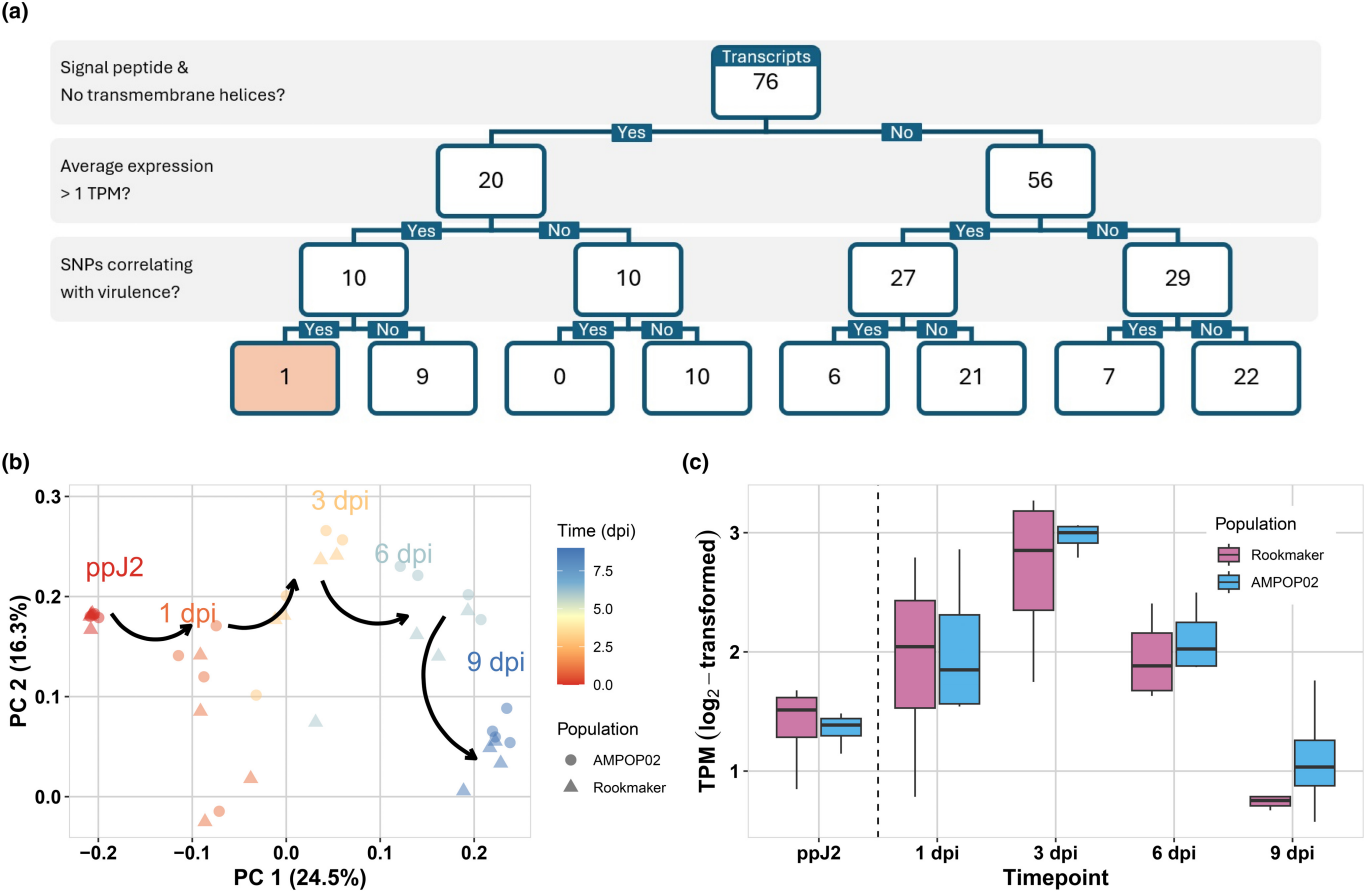
Gene *Gp‐pat‐1* meets all three criteria of a resistance‐breaking effector. (a) A flowchart of the three selection criteria leading to the identification of a single candidate (highlighted box). (b) Principal component (PC) analysis on RNA‐seq data of preparasitic juveniles (ppJ2) and parasitic juveniles shows that time captures most of the variance (24.5%). This indicates a transcriptional shift in the nematode as the infection progresses. (c) Expression of *Gp‐pat‐1* in the avirulent Rookmaker and virulent AMPOP02 populations on Seresta. Expression peaks at 3 d postinoculation, roughly coinciding with defence activation of *GpaVvrn*. Each box represents the interquartile range (Q1–Q3; IQR), the horizontal line inside the box marks the median, and the whiskers extend to the smallest and largest nonoutlier values (within 1.5× the IQR from the quartiles).

First, to assess which gene products are possibly secreted, we examined the 76 peptide sequences for the presence of an N‐terminal signal peptide for secretion and the absence of transmembrane helices. Based on these criteria, 20 of the 76 transcripts classified as putative effectors (Fig. 5a; Table S15).

Second, to assess the expression of the candidate genes during early stages of infection, an infection assay was performed on the *GpaV*_*vrn*_‐resistant potato variety Seresta. Preparasitic juveniles (ppJ2s) from avirulent (Rookmaker) and virulent (AMPOP02) *G. pallida* populations were inoculated on 2‐wk‐old potato cuttings. A subsample of ppJ2s was harvested at inoculation and infected root tissue was harvested at 1, 3, 6, and 9 dpi and subjected to transcriptome sequencing. On average, 77.7 million read pairs were generated per sample, with 87.5% of ppJ2 reads and 2.6% of reads from infected root tissue mapping to the *G. pallida* Rookmaker genome (Table S13). PC analysis revealed that within the dataset, time is the factor explaining most of the variance (Fig. 5b). Interestingly, none of the 76 transcripts showed statistically significant expression differences between Rookmaker and AMPOP02 at any timepoint during infection (FDR < 0.01). Given that *S. verneii* resistance has been described as a ‘late’ type of resistance response, activated in the first few days of infection, but not within the first 24 h (Rice *et al*., 1987), we hypothesised that the expression of a resistance‐breaking effector coincides with defence activation. Therefore, we prioritised candidates with an average log_2_‐transformed expression of at least 1.0 TPM at 3 dpi. Of the 76 transcripts, 37 transcripts passed this threshold (Table S16). Among the 20 putative effectors, 10 transcripts passed the threshold (Fig. 5a).

Third, we assessed allelic variation and alternative allele frequencies in AMPOP02 and AMPOP10 over the generations in the selection experiment. Since transcriptomic analysis did not indicate differences in expression levels between Rookmaker and AMPOP02, we inferred that virulence likely results from a structurally different effector, rather than a quantitative difference in expression. Therefore, we prioritised variants affecting the CDS of the genes in the region. Of the 76 transcripts at the locus, 14 contained SNPs positively and significantly (FDR < 0.01) correlating with virulence in AMPOP02 and AMPOP10 (Table S15). Among the 10 candidates, just 1 contained allelic variation (*Gpal_Rook_g6760.t1‐0004*). As this is the only gene passing all three selection criteria, this gene is considered the prime candidate for breaking *GpaVvrn* resistance. Since the gene was annotated with the GO term adenylyltransferase activity (GO:0070566), we named it *Gp‐pat‐1*.

Interestingly, *Gp‐pat‐1* also shows the most significant quadratic correlation with time of all putative effectors (Adj. *P*‐value = 9.53e‐7; Fig. 5c), suggesting that expression coincides with the *GpaV*_*vrn*_ defence response.

### Silencing *Gp‐pat‐1* enhances virulence on *GpaV*_*vrn*_ ‐resistant potato varieties

**Fig. 6:**
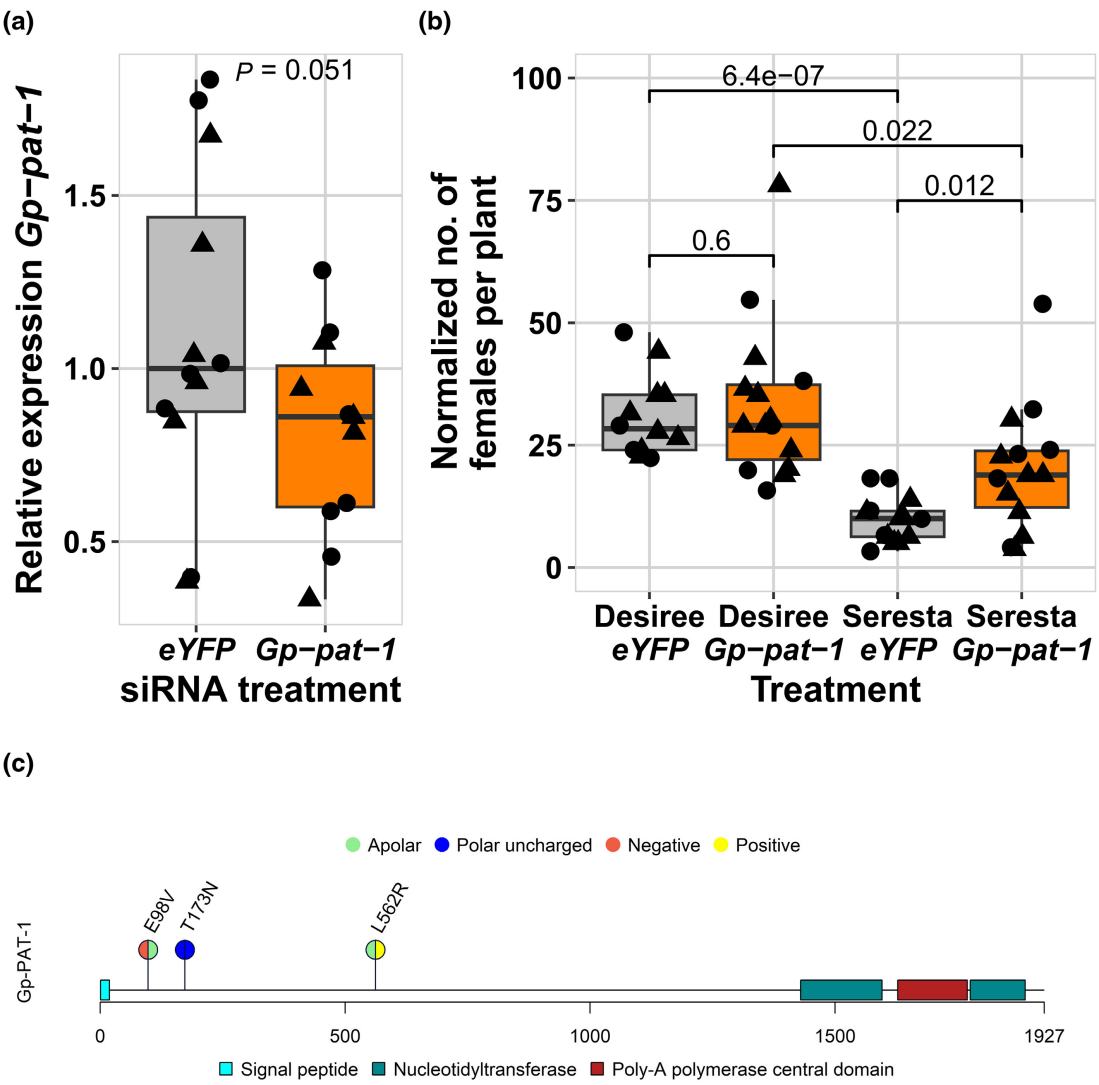
RNAi of *Gp‐pat‐1* increases *Globodera pallida* virulence on the *GpaVvrn*‐resistant potato variety Seresta. (a) Expression levels of *Gp‐pat‐1* at 3 d after inoculation. Each data point represents the *Gp‐pat‐1* expression in infected root tissue of 10–15 plants. Expression was relative to the median expression in *eYFP*‐treated ppJ2. Combined data from two batches and two potato varieties (Desiree and Seresta) are shown (*n* = 12); Batch 1 values as circles and Batch 2 values as triangles. Each box represents the interquartile range (Q1–Q3; IQR), the horizontal line inside the box marks the median, and the whiskers extend to the smallest and largest nonoutlier values (within 1.5× the IQR from the quartiles). The significance shown is based on a one‐sided *t*‐test. (b) Juveniles with altered *Gp‐pat‐1* expression were more virulent on the *GpaVvrn*‐resistant Seresta compared with the control juveniles that are soaked in *eYFP*‐targeting siRNAs (*n* = 14). Virulence of *Gp‐pat‐1*‐silenced juveniles (*n* = 15) is unchanged from *eYFP*‐treated juveniles (*n* = 12) on the susceptible Desiree variety. Combined data from two batches are shown: Batch 1 values as circles and Batch 2 values as triangles. Female counts were normalised per batch to the mean of the *eYFP*‐treated controls on Desiree and scaled to the overall mean of both batches for crossexperiment comparison. The *P*‐values shown are from two‐sided *t*‐tests. (c) Schematic representation of *Gp*‐PAT‐1 depicting the predicted functional domains and the four nonsynonymous single‐nucleotide polymorphisms (SNPs) that correlate significantly with virulence in AMPOP02 and AMPOP10. These four SNPs cause three amino acid changes. Colours of the circles indicate the polarity of the amino acid according to the reference sequence (left half) and the alternative sequence (right half).

To assess the role of our prime candidate gene in the breakdown of *GpaV*_*vrn*_ resistance, we silenced *Gp‐pat‐1* by soaking juveniles of AMPOP02 in small interfering RNAs (siRNAs; Table S4). Since the *Gp‐pat‐1* expression in preparasitic juveniles is not representative for the expression at 3 dpi (Fig. 5c), we used infected root tissue to assess expression levels. This resulted in a 0.72‐fold expression of our putative effector at 3 dpi (Fig. 6a). Although expression levels were overall lower, there was a lot of biological variation. Upon inoculating Desiree and Seresta, we observed a significant increase of virulence on Seresta, but not on Desiree (Fig. 6b). The unchanged virulence of *G. pallida* on the susceptible Desiree, combined with increased virulence on the *GpaV*_*vrn*_‐resistant Seresta, indicates that *Gp‐pat‐1* functions as an avirulence factor recognised by *GpaV*_*vrn*_.

To further characterise *Gp‐pat‐1*, we searched for functional domains using InterPro (Paysan‐Lafosse *et al*., 2023). This identified a poly‐A polymerase domain, which includes three subdomains: a central poly‐A polymerase domain flanked by two nucleotidyltransferase domains on both sides (Fig. 6c). According to InterPro predictions, the *Gp*‐PAT‐1 protein is located in the nucleus (GO:0005634), where it may be involved in poly‐A RNA polymerase activity (GO:1990817). *Gp‐pat‐1* is a single copy gene without any homologues on the *G. pallida* genome. The only nematode species with a *Gp‐pat‐1* homologue is *G. rostochiensis*. A BLASTp against the *G. rostochiensis* Line 19 and Line 22 genomes (van Steenbrugge *et al*., 2021) indicated that on both genomes *Gr‐pat‐1* was annotated as two separate genes (Gr19_g4522 and g4523; Gr22_g11715 and g11716) with pairwise identities of 67.6% and 60.8%, respectively. The best BLASTp hit on the *Heterodera schachtii* Bonn genome (*Hsc_gene_4407*) has 44.4% similarity (Siddique *et al*., 2022).

Since *Gp‐pat‐1* is not part of a known effector family and poly‐A polymerases are not known to be secreted, we assessed the secretory potential of poly‐A polymerases in cyst nematodes. In *G. pallida*, we found 8 of the 44 transcripts annotated with poly‐A RNA polymerase activity to have a predicted signal peptide for secretion, and in *H. schachtii* 4 out of 96 (Table S17). As stylet‐secreted effector proteins are known to be produced in the oesophageal glands, we assessed gland cell expression of putative *Heterodera schachtii* poly‐A polymerases with data previously published (Molloy *et al*., 2024). Interestingly, 68 out of 96 (71%) putative poly‐A polymerases showed expression (TPM > 1) in oesophageal gland cells of preparasitic J2s, parasitic J2s and/or parasitic J3s. This includes the best BLASTp hit of *Gp‐pat‐1* (*Hsc_gene_g4407*; Fig. S13). Together, this supports the hypothesis that putative poly‐A polymerases, such as *Gp‐pat‐1*, can be secreted into the host.

To identify variants within *Gp‐pat‐1* potentially causal for virulence, we assessed the allelic variants that significantly correlated with virulence in the selection experiment (FDR < 0.01). Within the CDS of *Gp‐pat‐1*, we identified four SNPs leading to nonsynonymous changes in the protein. As two of the SNPs were located within the same codon, these four SNPs caused three amino acid changes, two of which altered the charge of the amino acid (Fig. 6c). Over the five generations of the selection experiment, the average AAF of these four SNPs increased from 0.49 to 0.71, indicating an average AAF increase of 0.044 per generation. We therefore conclude that *Gp‐pat‐1* is a strong candidate effector associated with virulence on *GpaV*_*vrn*_.

## Discussion

### *Globodera pallida* virulence is the result of the widespread use of *GpaV*_*vrn*_

We show that resistance‐breaking *G. pallida* field populations are virulent on a broad range of potato varieties. Moreover, the level of virulence on one resistant variety correlated with the level of virulence on other resistant varieties. This suggests that virulence is the result of the breakdown of a common source of resistance present in all tested potato varieties. Our data identify *GpaV*_*vrn*_ as the major resistance broken by virulent *G. pallida* populations. The broad presence of *GpaV*_*vrn*_ is in line with the literature, wherein *GpaV*_*vrn*_ was expected to be the primary source of *S. vernei*‐derived resistance in French and British *G. pallida*‐resistant commercial potato varieties (Fournet *et al*., 2013; Varypatakis *et al*., 2019). Other reports have mentioned *Grp1* as the main source of *G. pallida* resistance in commercial potato varieties (Grenier *et al*., 2020). *Grp1* co‐localises with *GpaV*_*vrn*_ on Chromosome 5, but is not present in all varieties tested here and its breakdown is therefore unlikely to explain the increase in virulence observed here (van der Voort *et al*., 1998; Finkers‐Tomczak *et al*., 2009).

Cluster analysis on the susceptibility levels of 29 potato varieties divided the potato varieties into two clusters: Cl_SER_ and Cl_FES_, named after the often‐grown Seresta and Festien varieties. Resistance levels in Cl_FES_ correlate with – but exceed that of – Cl_SER_, suggesting that Cl_FES_ might have additional minor resistances to *G. pallida* on top of *GpaV*_*vrn*_. Candidates for this additional resistance include *Gpa6*, *Grp1, GpaV*_*spl*_, and *GpaXI*_*spl*_ (Rouppe van der Voort *et al*., 2000; Caromel *et al*., 2005; Gartner *et al*., 2021). However, our analyses are inconclusive as to the nature of the additional gene(s). Our data do point to a scenario of a broad application of *GpaV*_*vrn*_ since the introduction of the first potato varieties carrying *GpaV*_*vrn*_ in 1994 (Van Berloo *et al*., 2007). This narrow genetic basis of *GpaV*_*vrn*_ resistance, especially in the Cl_SER_, led to the emergence of virulent *G. pallida* field populations. Since adaptation of *G. pallida* populations to Cl_SER_ potato varieties also enhances their virulence on Cl_FES_ varieties, selection on *GpaV*_*vrn*_ not only breaks resistance in CL_SER_ but also increases the potential of these populations to overcome additional resistances present in Cl_FES_ varieties.

### Virulence is widespread and results from selection on standing variation

The origin of virulence alleles has big implications for their geographical distribution. When virulence alleles were present in *G. pallida* field populations before the introduction of *GpaV*_*vrn*_, virulence might be widely distributed, but if virulence alleles arose from recent *de novo* mutations, virulence likely is restricted to a single field or a limited set of neighbouring fields. We demonstrate widespread virulence across the northeast of the Netherlands and that differences in virulence levels reflect differences in stages of selection. Thus, we conclude that virulence is likely the result of selection on standing variation. Similar patterns of selection have been reported in other plant pathogens, such as the potato pathogen *Phytophthora infestans* (Morales *et al*., 2020) and the oat pathogen *Puccinia coranata* (Miller *et al*., 2020), where the deployment of resistance genes caused shifts in allele frequencies at avirulence loci.

*Globodera pallida* virulence is not limited to the Netherlands. The parallel emergence of German resistance‐breaking populations (Niere *et al*., 2014) and the resistance‐breaking potential of French and English populations (Phillips & Blok, 2008; Fournet *et al*., 2013; Varypatakis *et al*., 2019) indicate that virulence alleles are present in *G. pallida* populations throughout Western Europe. The spread of virulence across Europe suggests that virulence alleles were already present in the founding populations that were introduced into Europe roughly 150 yr ago (Plantard *et al*., 2008). This is supported by the observation that some Peruvian *G. pallida* populations are virulent on *GpaV*_*vrn*_, without being exposed to this resistance in Europe (Hockland *et al*., 2012). Given that all European mainland *G. pallida* populations originated from a single historical introduction (Plantard *et al*., 2008; Grenier *et al*., 2020), it is also unlikely that virulence alleles were introduced into Europe in a more recent undescribed introduction of *G. pallida* into Europe. Since all European mainland populations are from the same historic introduction, and the build‐up of virulence observed in our selection experiment is comparable to the virulence build‐up in other artificial selection experiments with *G. pallida* on *GpaV*_*vrn*_ (Fournet *et al*., 2013; Varypatakis *et al*., 2019), likely the same allele is underlying virulence on *GpaV*_*vrn*_ in each of these *G. pallida* populations. To test whether a shared virulence allele is responsible for virulence across Western Europe, future research should compare the genetic basis of virulence in Dutch populations with that of other recent West‐European *G. pallida* populations.

### Associating virulence to a single locus suggests a gene‐for‐gene interaction

Given that virulence results from a single selection pressure in potato, we hypothesised that virulence results from selection on a single *G. pallida* locus. Studies on other nematode species suggest that heritabilities within the range we observed (*H* ^2^ ≥ 0.73) are often associated with a single genetic locus (Evans *et al*., 2021). High‐coverage whole‐genome sequencing on each generation of the two selected populations and the assembly of the new highly contiguous *G. pallida* Rookmaker reference genome allowed us to associate virulence to a single 311 Kb locus. In comparison, two previous genome scans on French *G. pallida* populations virulent on *GpaV*_*vrn*_ landed on genomic regions of roughly 2 Mb scattered over multiple scaffolds (Eoche‐Bosy *et al*., 2017a, 2017b). A third genome scan on British *S. vernei‐*selected *G. pallida* populations combined two variant calling analyses, PenSeq and ReSeq, resulting in the identification of 4 *S. vernei*‐associated genes (Varypatakis *et al*., 2020). However, none of these genes is located within our virulence locus. Interestingly, a recent fourth genome scan on two French *GpaV*_*vrn*_‐selected *G. pallida* populations converged on two genomic loci, including a locus towards the end of scaffold 2 of the D383 genome (Lechevalier *et al*., 2025a). Wince this matches the virulence locus identified by our association analysis it provides independent support that this locus is involved in the breakdown of *GpaV*_*vrn*_.

Linking virulence to a single locus suggests that virulence on *GpaV*_*vrn*_ follows the classical gene‐for‐gene model (Flor, 1971), in which an effector with avirulence activity triggers a resistance‐specific defence response, leading to plant immunity. This model has been widely used for informed resistance deployment and also applies to cyst nematodes (Janssen *et al*., 1991). In the future, identifying the *GpaV*_*vrn*_ gene from the nine reported candidates (Wang *et al*., 2023) will enable testing whether *GpaV*_*vrn*_ and its corresponding avirulence effector conform to the gene‐for‐gene model. The rapid adaptation of *G. pallida* to *GpaV*_*vrn*_ illustrates how reliance on a single major resistance can result in its breakdown. Combining or rotating resistance sources may be necessary for durable control of *G. pallida* in the field.

### The novel effector *Gp‐pat‐1* has avirulence activity on *GpaV*_*vrn*_

We identified variation in the *Gp‐pat‐1* gene as the cause of (a)virulence on *GpaV*_*vrn*_‐resistant potato plants. Our finding builds on the assumption that stylet‐secreted effectors of *G. pallida* are the most likely candidates underlying avirulence (Mitchum *et al*., 2013). Therefore, we only focused on genes encoding proteins containing a predicted signal peptide and lacking a transmembrane helix. Furthermore, we based our candidate gene search on expression levels and the presence of genetic variation. Here, it proved to be crucial to work with accurate, manually curated gene models (Moya *et al*., 2023). The expression of *Gp‐pat‐1* upon host infestation aligns with typical effector expression patterns, where infective juveniles utilise effectors to migrate through the root, to establish the syncytium, and to suppress host immune responses (Gardner *et al*., 2015; Da Rocha *et al*., 2021; Wen *et al*., 2021; Chen *et al*., 2023).

To assess avirulence activity associated with *Gp‐pat‐1* on *GpaV*_*vrn*_‐resistant potato varieties, we silenced the gene in preparasitic second stage juveniles of *G. pallida* AMPOP02. Silencing of *Gp‐pat‐1* increased the number of juveniles that developed into mature females on Seresta, which contains *GpaV*_*vrn*_, but not on the susceptible Desiree. This indicates that in this population the gene has avirulence activity on *GpaV*_*vrn*_. To assess whether silencing *Gp‐pat‐1* enhances virulence, future research can test other virulent populations, such as our AMPOP10 population or the German Oberlangen population (Mwangi *et al*., 2019b). Since silencing resulted in increased virulence, this suggests that either the protein itself or its effects are recognised by *GpaV*_*vrn*_. Future research can evaluate this hypothesis by *Agrobacterium*‐mediated transient expression of different *Gp‐pat‐1* alleles in a *GpaV*_*vrn*_ background. We did not observe a fitness cost upon silencing *Gp‐pat‐1* in a susceptible background. This is in line with previous research showing no fitness costs for virulence on *GpaV*_*vrn*_ (Fournet *et al*., 2016) and could be either due to a biological factor, such as genetic redundancy, or a technical limitation, such as insufficient resolution or statistical power.

Although the role of *Gp*‐PAT‐1 in the host was beyond the scope of this study, *in silico* analysis provided first insights into protein functioning. Domain prediction identified the domains of a poly‐A polymerase and the subcellular localisation in the nucleus. To date, no nematode effector has been reported to possess nucleotidyltransferase activity. By contrast, several bacterial plant pathogen effectors exhibit such activity, including the *Xanthomonas* effector XopAC (Feng *et al*., 2012). Although we have no evidence that *Gp‐pat‐1* is a stylet‐secreted effector, the secretory potential of *Gp‐pat‐1* is supported by the observation that the majority (71%) of putative poly‐A polymerases in the cyst nematode *H. schachtii* were expressed in the glands, from where they may be secreted into the host. As incorrect polyadenylation affects translation, *Gp‐*PAT‐1 may be secreted to interfere with transcriptional regulation of the host.

### Conclusion

We show that selection on *GpaV*_*vrn*_‐mediated resistance is responsible for the current outbreak of virulence in *G. pallida* field populations in the Netherlands. By associating virulence to a single locus in *G. pallida* and identifying a single gene involved in overcoming *GpaV*_*vrn*_ resistance, our findings open avenues to develop molecular diagnostic tools to monitor *G. pallida* virulence in the field. Our data suggested that selection by *GpaV*_*vrn*_ on standing genetic variation led to the emergence of virulence in *G. pallida*. However, testing this hypothesis requires molecular investigations on historic populations and multiple virulent populations. If virulence has a shared molecular basis, monitoring virulence allele frequencies will allow for the early detection of virulence build‐up in the field and will allow farmers to make informed agronomical decisions. To ultimately control these virulent *G. pallida* populations, novel resistance genes must be introduced into commercial potato varieties and used strategically.

## Competing interests

This research was executed as part of a public/private partnership project funded by the Dutch government, including co‐financing from several public and private organisations. The authors declare that the research was conducted in the absence of any commercial or financial relationships that could be construed as a potential conflict of interest.

## Author contributions

ASS, MGS and GS designed the experiments. CCvS and SvdE assisted in DNA isolation. PH conducted the selection experiments. JJMvS, DMtM, SJSvdR, ASS and MGS conducted the genetic analyses and assembled and annotated the Rookmaker genome. *In vitro* infection assays for RNA‐seq and functional validation were conducted by ASS. ASS, MGS and GS wrote the paper with input from all other co‐authors.

## Disclaimer

The New Phytologist Foundation remains neutral with regard to jurisdictional claims in maps and in any institutional affiliations.

## Supporting information


**Fig. S1** Read‐depth histogram used for haplotig purging.
**Fig. S2** The relative susceptibilities of 16 potato varieties towards seven *Globodera pallida* populations as measured in the first standard PCN resistance test.
**Fig. S3** The relative susceptibility and propagation of nine virulent *Globodera pallida* field populations on 28 potato varieties.
**Fig. S4** Three clusters of resistant potato varieties explain most of the variance in propagation of virulent *Globodera pallida* populations.
**Fig. S5** Small container test data shows strong correlation with the data from the second and third standard PCN resistance tests.
**Fig. S6** Variance in virulence between *Globodera pallida* populations follows a gradient without clear clustering.
**Fig. S7** The reproductive properties of the two *Globodera pallida* selection populations on six potato varieties.
**Fig. S8** Identification of the Seresta‐selected loci in *Globodera pallida* populations AMPOP02 and AMPOP10 based on the *G. pallida* Rookmaker genome.
**Fig. S9** Analysis for variants on the *Globodera pallida* Rookmaker genome associated with generation.
**Fig. S10** Synteny plot between the *Globodera pallida* D383 genome and the *G. pallida* Rookmaker genome.
**Fig. S11** A region associated with virulence on GpaV_*vrn*_, syntenic to *Globodera pallida* Rookmaker Scaffold 28 was identified on the D383 genome.
**Fig. S12** Region on scaffold 28 of the *Globodera pallida* Rookmaker genome associated with virulence.
**Fig. S13** Gland cell expression of the *Heterodera schachtii* gene *Hsc_gene_g4407* across three distinct stages.
**Note S1** Results of the manual genome annotation of the avirulence locus.

**Table S1** Overview of all 31 potato varieties tested across four PCN resistance tests.
**Table S2** Data of the fourth resistance pot tests.
**Table S3** An overview of the RNA samples used for temporal transcriptome analysis.
**Table S4** RNA and DNA oligonucleotides used for RT‐qPCR.
**Table S5** Data of the first resistance pot test.
**Table S6** Data of the second and third resistance pot tests.
**Table S7** The variants linked to the *GpaV*_*vrn*_ locus as identified by graphical mapping using Desiree and Innovator as references.
**Table S8** The variants linked to the *Gpa6* locus as identified by graphical mapping using Desiree and Festien as references.
**Table S9** Data of the small container tests.
**Table S10** Analysis of variance for potato variety cluster and *Globodera pallida* field population virulence.
**Table S11** Heritability analyses on the fourth standard potato cyst nematode (PCN) resistance test data, selection experiment populations and the small container test data.
**Table S12** An overview of the DNA sequenced samples of the *Globodera pallida* selection experiment.
**Table S13** Comparative genome statistics of the *Globodera pallida* Rookmaker genome assembly from the current paper and the D383 genome (van Steenbrugge *et al*., 2023).
**Table S14** The significantly associated variants in *Globodera pallida* with increases and decreases in alternative allele frequencies over generations.
**Table S15** An overview of the 76 manually annotated transcripts on the *Globodera pallida* avirulence locus.
**Table S16** Transcription levels of each *Globodera pallida* transcript at each of the five time points measured.
**Table S17** Poly‐A polymerases in *Heterodera schachtii* and *Globodera pallida*.
Please note: Wiley is not responsible for the content or functionality of any Supporting Information supplied by the authors. Any queries (other than missing material) should be directed to the *New Phytologist* Central Office.

## Data Availability

All scripts and underlying datasets are available through gitlab (https://git.wur.nl/published_papers/Schaveling_2025_Pallifit_virulence). The data of presented resistance tests have been included Tables S2, S5, S6, S9. The DNA sequencing data of the selection experiment are deposited at BioStudies (E‐MTAB‐15408). The Rookmaker genome, the short‐ and long‐read Rookmaker DNA sequencing data (ERR15233941 and ERR15205749, respectively), the structural annotation, and the RNA‐sequencing data used for structurally annotating the Rookmaker genome (ERR15277786) were deposited at the ENA with accession no.: PRJEB91928. RNA‐seq of the infection assay presented in this paper was deposited at BioStudies (E‐MTAB‐15312).
