# Supplementary material for: The potato cyst nematode Globodera pallida overcomes major potato resistance through selection on standing variation at a single locus
Source: New Phytol. 2026 Jan 6;249(6):3039–59. doi: 10.1111/nph.70886 (PMC12917471; doi:10.1111/nph.70886)
Supplement: Supplementary file 1 — Fig. S1 Read‐depth histogram used for haplotig purging. Fig. S2 The relative susceptibilities of 16 potato varieties towards seven Globodera pallida populations as measured in the first standard PCN resistance test. Fig. S3 The relative susceptibility and propagation of nine virulent Globodera pallida field populations on 28 potato varieties. Fig. S4 Three clusters of resistant potato varieties explain most of the variance in propagation of virulent Globodera pallida populations. Fig. S5 Small container test data shows strong correlation with the data from the second and third standard PCN resistance tests. Fig. S6 Variance in virulence between Globodera pallida populations follows a gradient without clear clustering. Fig. S7 The reproductive properties of the two Globodera pallida selection populations on six potato varieties. Fig. S8 Identification of the Seresta‐selected loci in Globodera pallida populations AMPOP02 and AMPOP10 based on the G. pallida Rookmaker genome. Fig. S9 Analysis for variants on the Globodera pallida Rookmaker genome associated with generation. Fig. S10 Synteny plot between the Globodera pallida D383 genome and the G. pallida Rookmaker genome. Fig. S11 A region associated with virulence on GpaV vrn , syntenic to Globodera pallida Rookmaker Scaffold 28 was identified on the D383 genome. Fig. S12 Region on scaffold 28 of the Globodera pallida Rookmaker genome associated with virulence. Fig. S13 Gland cell expression of the Heterodera schachtii gene Hsc_gene_g4407 across three distinct stages. Note S1 Results of the manual genome annotation of the avirulence locus. [file NPH-249-3039-s001.pdf]

New Phytologist Supporting Information

Article title: The potato cyst nematode *Globodera pallida* overcomes major potato resistance through selection on standing variation at a single locus

Authors: Arno S. Schaveling, Dennie M. te Molder, Paul Heeres, Joris J.M. van Steenbrugge, Stefan J.S. van de Ruitenbeek, Casper C. van Schaik, Sven van den Elsen, Geert Smant, Mark G. Sterken

Article acceptance date: 12 December 2025

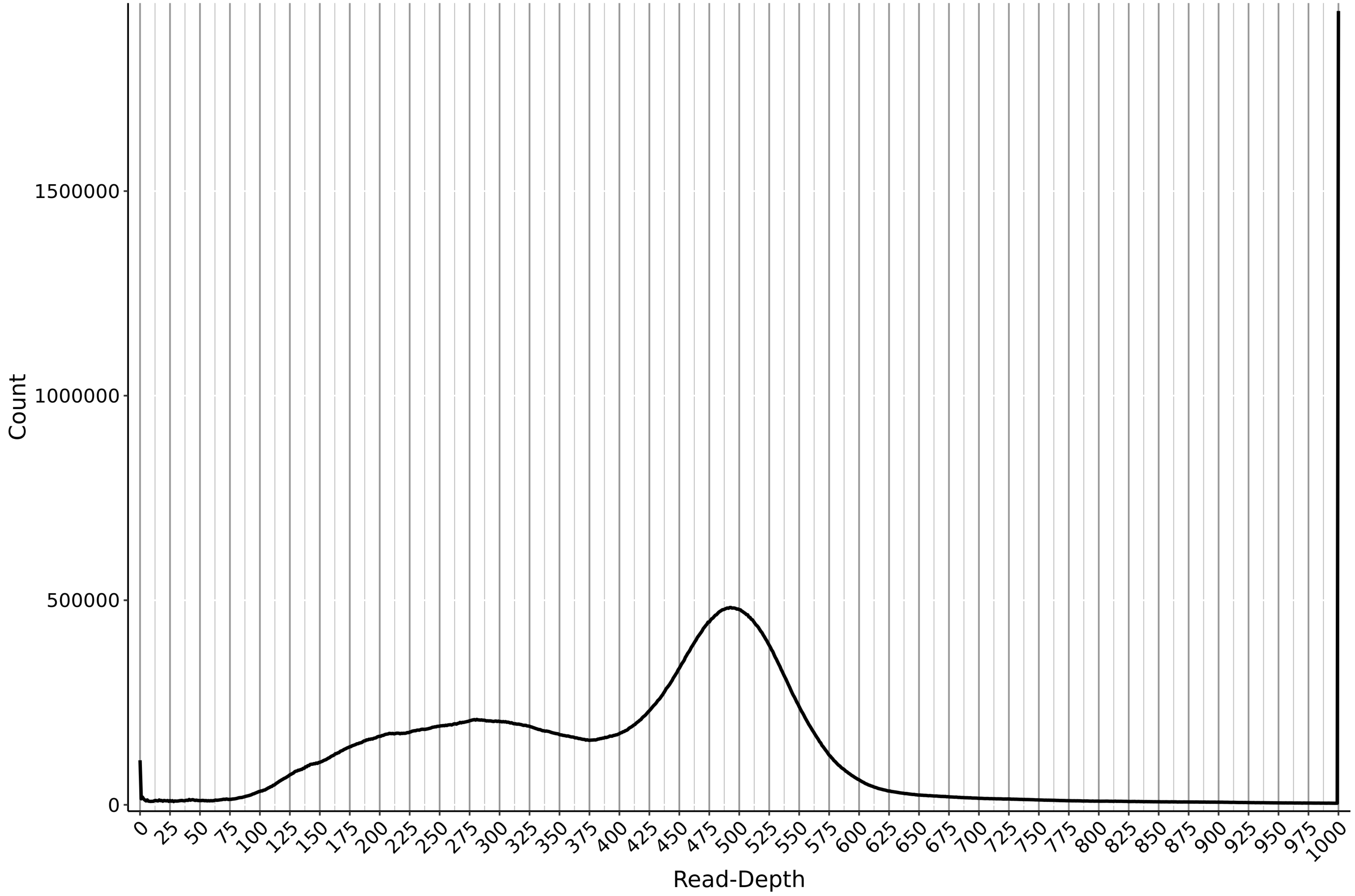

Count
1500000
1000000
500000
0
0
25
50
75
100
125
150
175
200
225
250
275
300
325
350
375
400
425
450
475
500
525
550
575
600
625
650
675
700
725
750
775
800
825
850
875
900
925
950
975
1000
Read-Depth

Fig. S1: Read-depth histogram used for haplotig purging. Histogram of Nanopore read-depth frequencies for the *Globodera pallida* draft assembly. The distribution shows a haploid peak and diploid peak, enabling selection of the depth thresholds used for haplotig removal.

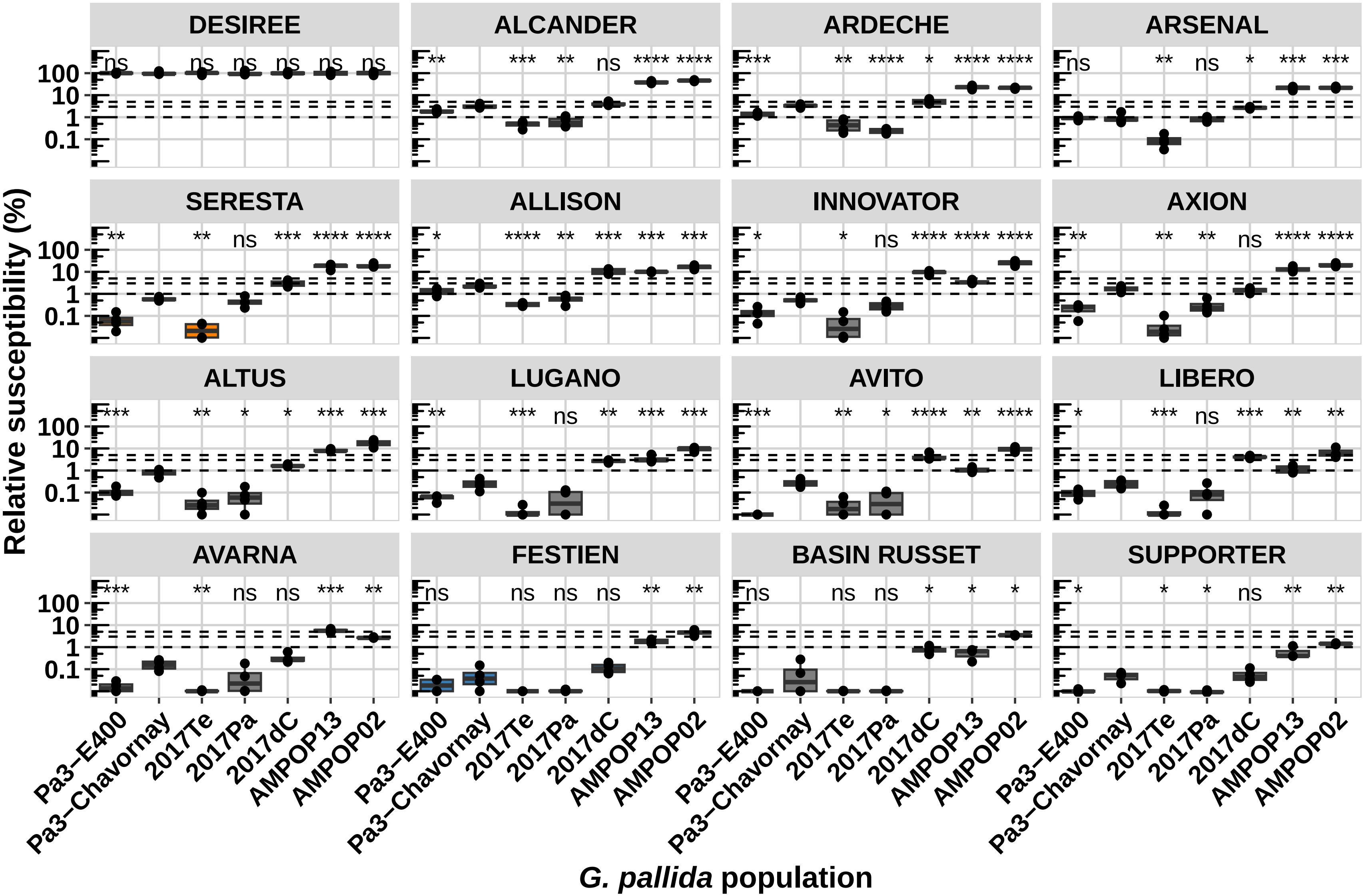
DESIREE
ALCANDER
ARDECHE
ARSENAL
SERESTA
ALLISON
INNOVATOR
AXION
ALTUS
LUGANO
AVITO
LIBERO
AVARNA
FESTIEN
BASIN RUSSET
SUPPORTER
Relative susceptibility (%)
100
10
1
0.1
Pa3–E400
Pa3–Chavornay
2017Te
2017Pa
2017dC
AMPOP13
AMPOP02
G. pallida population

Fig. S2: The relative susceptibilities of 16 potato varieties towards seven *Globodera pallida* populations as measured in the first standard potato cyst nematode (PCN) resistance test. Three field populations isolated from infection foci in fields with PCN resistant potato varieties (2017dC, AMPOP13, and AMPOP02) and two field populations without suspicions of virulence (2017Te and 2017Pa) were tested versus the standard population 'Chavornay'. The Dutch standard E400 'Rookmaker' is also included. The significances shown are from a two-sided t-test versus 'Chavornay' (* $p < 0.05$, ** $p < 0.01$, *** $p < 0.001$, **** $p < 0.0001$, ns = non-significant). Each box represents the interquartile range (Q1–Q3; IQR), the horizontal line inside the box marks the median, and the whiskers extend to the smallest and largest non-outlier values (within 1.5× the IQR from the quartiles).

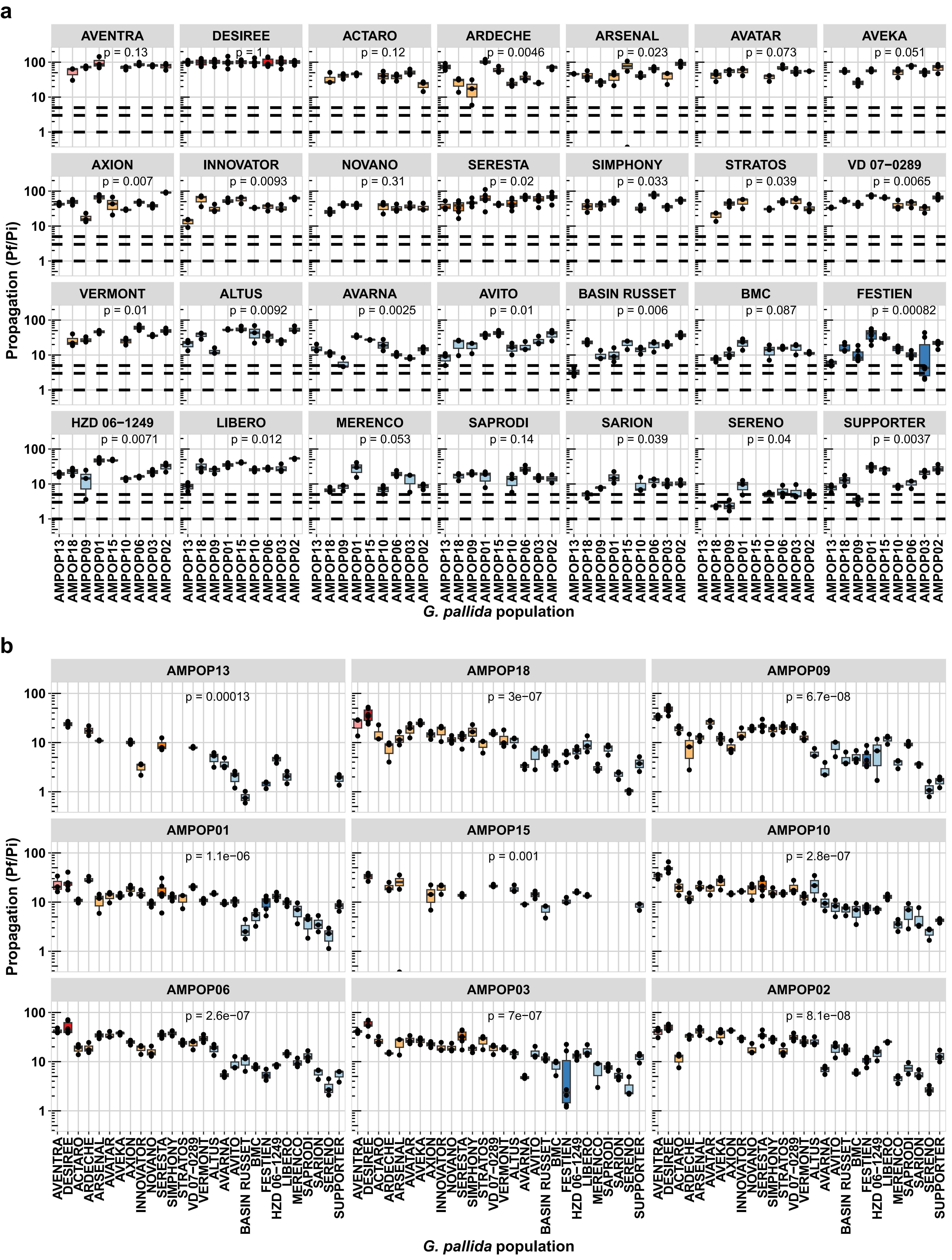
a
AVENTRA p = 0.13
DESIREE p = 1
ACTARO p = 0.12
ARDECHE p = 0.0046
ARSENAL p = 0.023
AVATAR p = 0.073
AVEKA p = 0.051
AXION p = 0.007
INNOVATOR p = 0.0093
NOVANO p = 0.31
SERESTA p = 0.02
SIMPHONY p = 0.033
STRATOS p = 0.039
VD 07−0289 p = 0.0065
VERMONT p = 0.01
ALTUS p = 0.0092
AVARNA p = 0.0025
AVITO p = 0.01
BASIN RUSSET p = 0.006
BMC p = 0.087
FESTIEN p = 0.00082
HZD 06−1249 p = 0.0071
LIBERO p = 0.012
MERENCO p = 0.053
SAPRODI p = 0.14
SARION p = 0.039
SERENO p = 0.04
SUPPORTER p = 0.0037
Propagation (Pf/Pi)
100
10
1
AMPOP13 AMPOP18 AMPOP09 AMPOP01 AMPOP15 AMPOP10 AMPOP06 AMPOP03 AMPOP02
G. pallida population
b
AMPOP13 p = 0.00013
AMPOP18 p = 3e−07
AMPOP09 p = 6.7e−08
AMPOP01 p = 1.1e−06
AMPOP15 p = 0.001
AMPOP10 p = 2.8e−07
AMPOP06 p = 2.6e−07
AMPOP03 p = 7e−07
AMPOP02 p = 8.1e−08
Propagation (Pf/Pi)
100
10
1
AVENTRA DESIREE ACTARO ARDECHE ARSENAL AVATAR AVEKA AXION INNOVATOR NOVANO SERESTA SIMPHONY STRATOS VD 07−0289 VERMONT ALTUS AVARNA AVITO BASIN RUSSET BMC FESTIEN HZD 06−1249 LIBERO MERENCO SAPRODI SARION SERENO SUPPORTER
G. pallida population

Fig. S3: The relative susceptibility and propagation of nine virulent *Globodera pallida* field populations on 28 potato varieties. (**a**) A boxplot of the relative susceptibility of the field populations per potato variety as determined in a second and third standard potato cyst nematode (PCN) resistance tests. Each dot represents a replicate experiment. The colours of the boxplots indicate to which cluster the potato varieties belong (**Fig. S4**), red for Cl_{DES}, orange for Cl_{SER}, and blue for Cl_{FES}. The p-values shown are from a kruskall-wallis test. When significant, the tests indicate there is between-population variation for the relative susceptibility. The *G. pallida* populations are ordered based on the clustering analysis from least to most virulent (**Fig. S5**). Each box represents the interquartile range (Q1–Q3; IQR), the horizontal line inside the box marks the median, and the whiskers extend to the smallest and largest non-outlier values (within 1.5× the IQR from the quartiles). (**b**) The propagation of the nine *G. pallida* populations on the 28 potato varieties. Colours and significance test as in (**a**). When significant, the tests indicate there is between-variety variation in propagation.

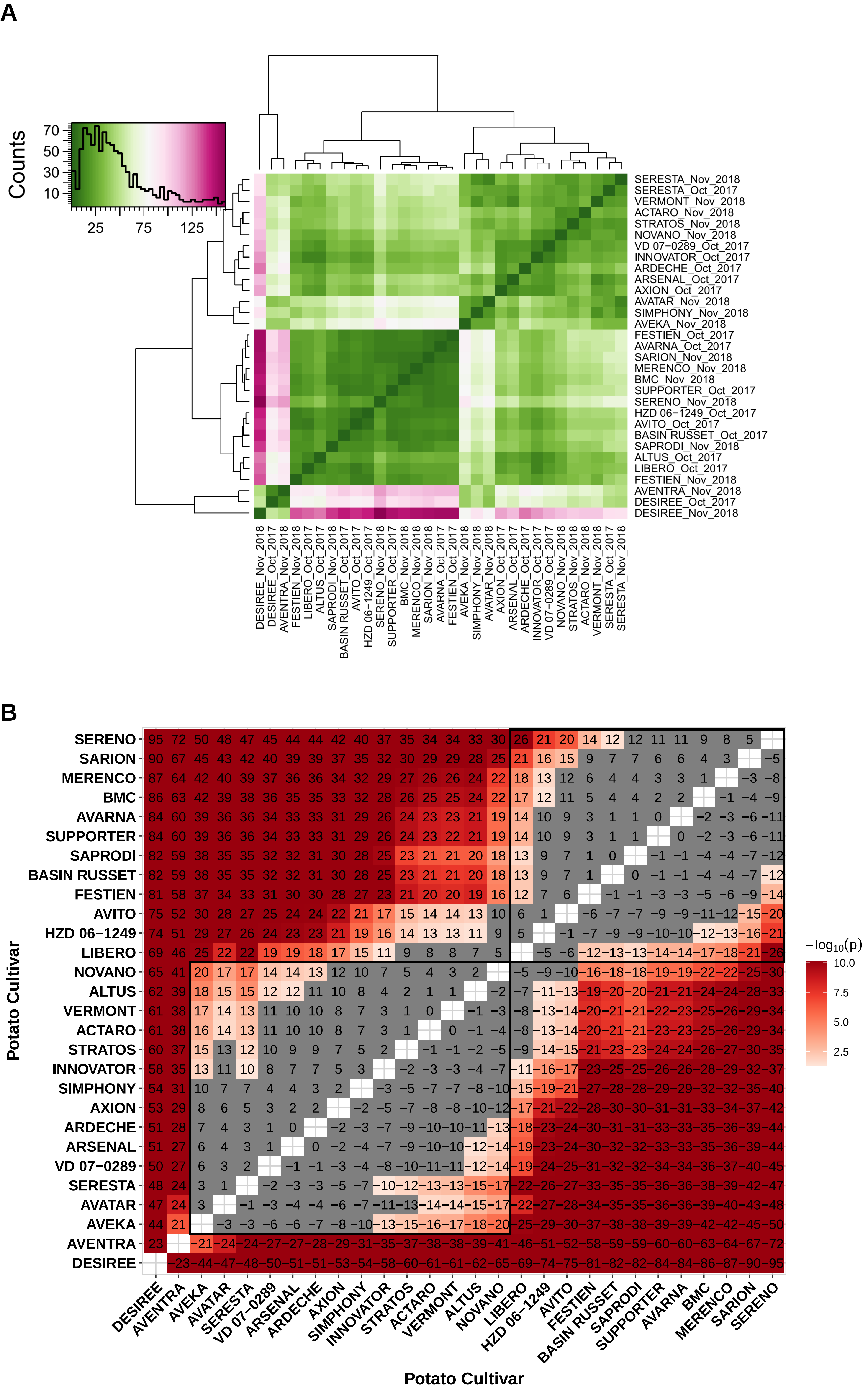

A
Counts
70
50
30
10
25
75
125
SERESTA_Nov_2018
SERESTA_Oct_2017
VERMONT_Nov_2018
ACTARO_Nov_2018
STRATOS_Nov_2018
NOVANO_Nov_2018
VD 07−0289_Oct_2017
INNOVATOR_Oct_2017
ARDECHE_Oct_2017
ARSENAL_Oct_2017
AXION_Oct_2017
AVATAR_Nov_2018
SIMPHONY_Nov_2018
AVEKA_Nov_2018
FESTIEN_Oct_2017
AVARNA_Oct_2017
SARION_Nov_2018
MERENCO_Nov_2018
BMC_Nov_2018
SUPPORTER_Oct_2017
SERENO_Nov_2018
HZD 06−1249_Oct_2017
AVITO_Oct_2017
BASIN RUSSET_Oct_2017
SAPRODI_Nov_2018
ALTUS_Oct_2017
LIBERO_Oct_2017
FESTIEN_Nov_2018
AVENTRA_Nov_2018
DESIREE_Oct_2017
DESIREE_Nov_2018
B
Potato Cultivar
SERENO
SARION
MERENCO
BMC
AVARNA
SUPPORTER
SAPRODI
BASIN RUSSET
FESTIEN
AVITO
HZD 06−1249
LIBERO
NOVANO
ALTUS
VERMONT
ACTARO
STRATOS
INNOVATOR
SIMPHONY
AXION
ARDECHE
ARSENAL
VD 07−0289
SERESTA
AVATAR
AVEKA
AVENTRA
DESIREE
−log10(p)
10.0
7.5
5.0
2.5
Potato Cultivar

Fig. S4: Three clusters of resistant potato varieties explain most of the variance in propagation of virulent *Globodera pallida* populations. (**a**) Euclidean clustering of the potato varieties based on the propagation levels of nine *G. pallida* field populations on 28 potato varieties. The data was obtained from the second (indicated with Oct_2017) and third (indicated with Oct_2018) standard potato cyst nematode (PCN) resistance tests. The clustering reveals three major clusters, ordered on the amount of propagation observed. The highest propagation is observed on non-resistant Desiree and Aventra (Cl_{DES}), subsequently there is a cluster containing Seresta (Cl_{SER}) and a cluster containing Festien (Cl_{FES}). Note that the placement of the Cl_{FES} in the middle is arbitrary. (**b**) The side-by-side differences between the varieties in relative susceptibility were tested using a TukeyHSD test, which was corrected for multiple testing. The numbers indicate the difference in relative susceptibility between the variety on the x-axis with the variety on the y-axis. For instance, the relative susceptibility in Aventra is 23% lower than Desiree. The colours indicate the $-\log_{10}(p)$ significance, where grey indicates there was no significant difference.

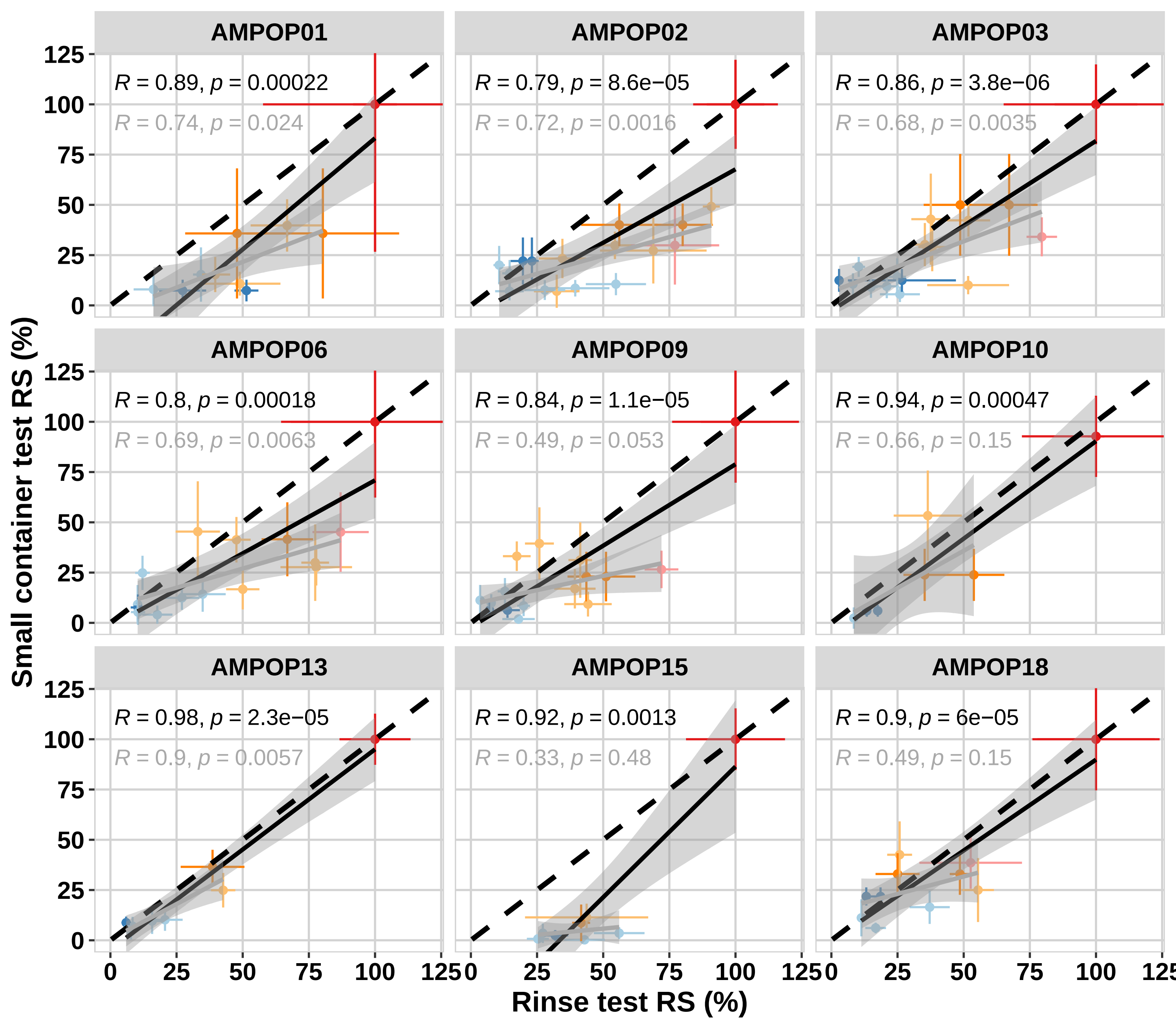
AMPOP01
R = 0.89, p = 0.00022
R = 0.74, p = 0.024
AMPOP02
R = 0.79, p = 8.6e−05
R = 0.72, p = 0.0016
AMPOP03
R = 0.86, p = 3.8e−06
R = 0.68, p = 0.0035
AMPOP06
R = 0.8, p = 0.00018
R = 0.69, p = 0.0063
AMPOP09
R = 0.84, p = 1.1e−05
R = 0.49, p = 0.053
AMPOP10
R = 0.94, p = 0.00047
R = 0.66, p = 0.15
AMPOP13
R = 0.98, p = 2.3e−05
R = 0.9, p = 0.0057
AMPOP15
R = 0.92, p = 0.0013
R = 0.33, p = 0.48
AMPOP18
R = 0.9, p = 6e−05
R = 0.49, p = 0.15
Small container test RS (%)
Rinse test RS (%)
0
25
50
75
100
125

Fig. S5: Small container test data shows strong correlation with the data from the second and third standard potato cyst nematode (PCN) resistance tests. The relative susceptibilities obtained from the nine virulent *Globodera pallida* field populations in the standard PCN resistance test were correlated with relative susceptibilities obtained from small container tests. The number of replicates for the standard PCN resistance test was n=3, for the small container tests the median was 8 replicates (between 7 and 14). Each dot represents the mean values for a variety and the horizontal and vertical lines represent the standard deviations. The colours indicate whether the variety belonged to the CL_{DES} (red), Cl_{SER} (orange), or CL_{FES} (blue). Desiree, Seresta, and Festien are coloured in bright red, bright orange, and bright blue, respectively. Two Pearson correlations were calculated, one including Desiree (on top, solid black line) and one excluding Desiree (second line, solid grey line). The dashed black line is added as a visual aid (equal relative susceptibilities from both tests).

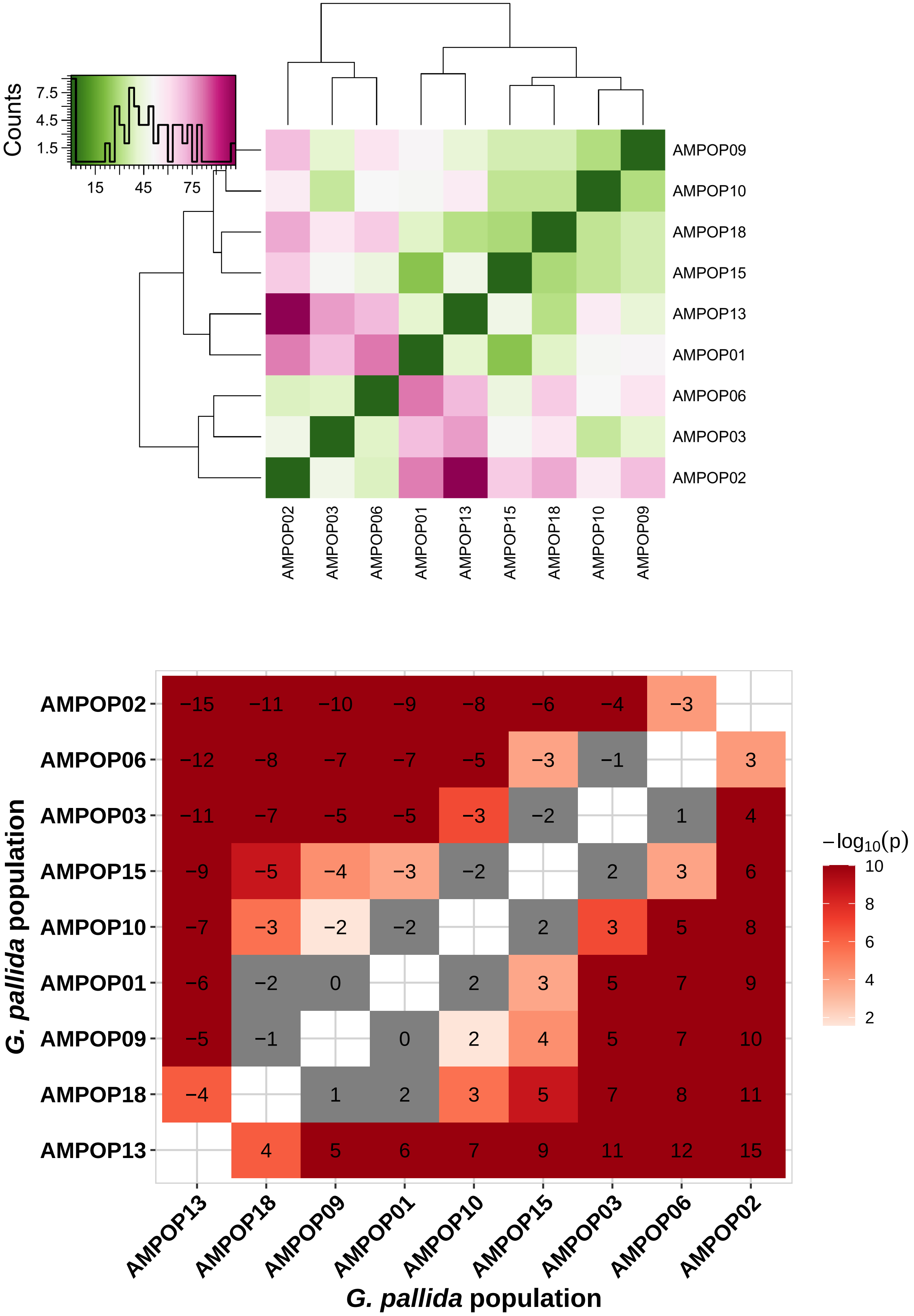
Counts
7.5
4.5
1.5
15
45
75
AMPOP09
AMPOP10
AMPOP18
AMPOP15
AMPOP13
AMPOP01
AMPOP06
AMPOP03
AMPOP02
AMPOP02
AMPOP03
AMPOP06
AMPOP01
AMPOP13
AMPOP15
AMPOP18
AMPOP10
AMPOP09
AMPOP02 −15 −11 −10 −9 −8 −6 −4 −3
AMPOP06 −12 −8 −7 −7 −5 −3 −1 3
AMPOP03 −11 −7 −5 −5 −3 −2 1 4
AMPOP15 −9 −5 −4 −3 −2 2 3 6
AMPOP10 −7 −3 −2 −2 2 3 5 8
AMPOP01 −6 −2 0 2 3 5 7 9
AMPOP09 −5 −1 0 2 4 5 7 10
AMPOP18 −4 1 2 3 5 7 8 11
AMPOP13 4 5 6 7 9 11 12 15
AMPOP13
AMPOP18
AMPOP09
AMPOP01
AMPOP10
AMPOP15
AMPOP03
AMPOP06
AMPOP02
G. pallida population
G. pallida population
−log10(p)
10
8
6
4
2

Fig. S6: Variance in virulence between *Globodera pallida* populations follows a gradient without clear clustering. (**a**) Euclidean clustering of the virulent *G. pallida* populations based on the propagation levels of nine *G. pallida* field populations on 28 potato varieties. The clustering reveals a gradient of virulence, from most virulent to least virulent. Note that the ordering of populations is arbitrary. (**b**) The side-by-side differences between the *G. pallida* field populations in propagation were tested using a Tukey HSD test, which was corrected for multiple testing. The numbers indicate the difference in propagation between the *G. pallida* field population on the x-axis with the *G. pallida* field population on the y-axis. For instance, the AMPOP02 has a Pf/Pi ratio that is on average 15 higher than that of AMPOP13. The colours indicate the $-\log_{10}(p)$ significance, where grey indicates there was no significant difference.

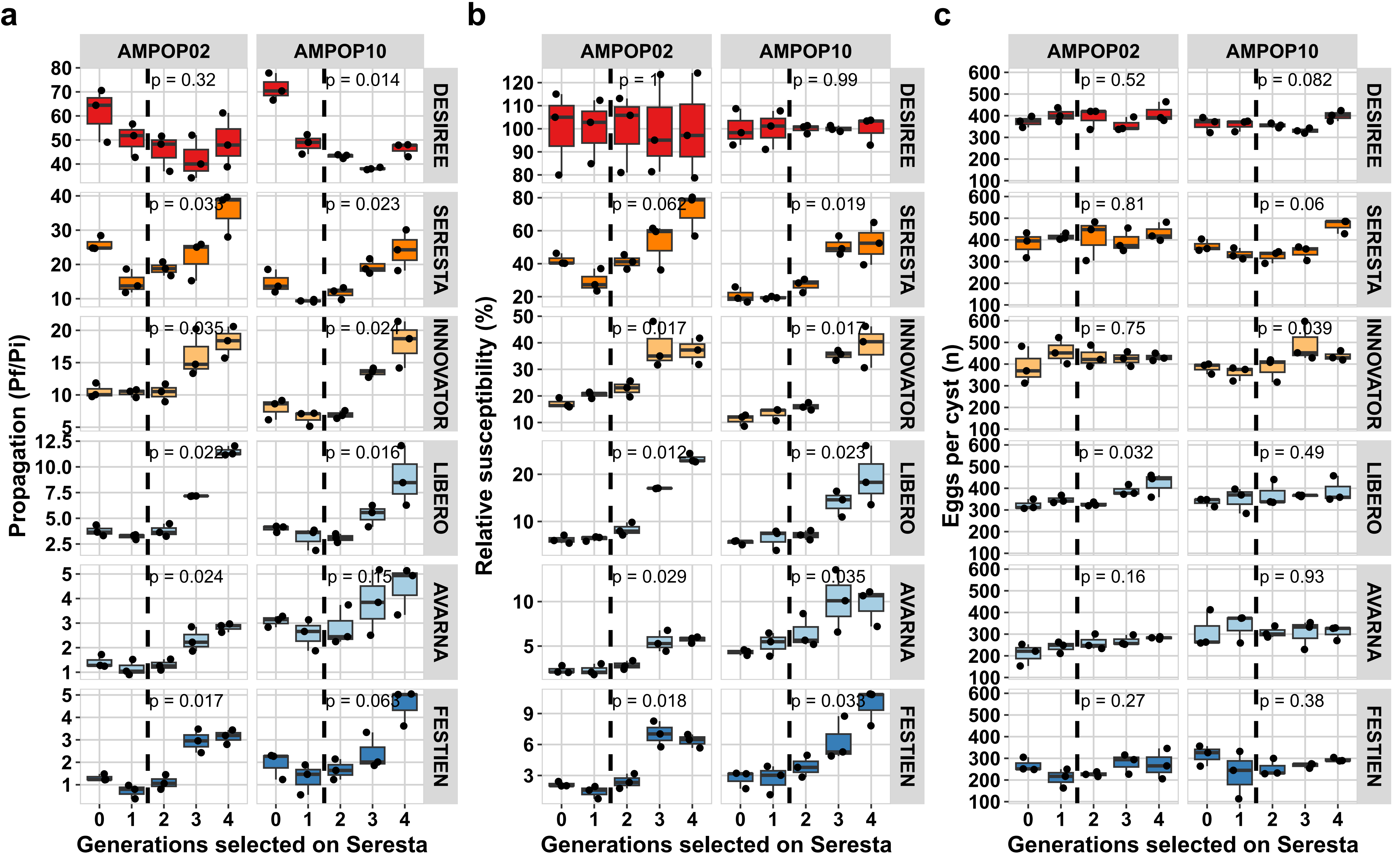
a
b
c
AMPOP02
AMPOP10
DESIREE
SERESTA
INNOVATOR
LIBERO
AVARNA
FESTIEN
Propagation (Pf/Pi)
Relative susceptibility (%)
Eggs per cyst (n)
Generations selected on Seresta
p = 0.32
p = 0.014
p = 1
p = 0.99
p = 0.52
p = 0.082
p = 0.062
p = 0.019
p = 0.81
p = 0.06
p = 0.017
p = 0.75
p = 0.039
p = 0.012
p = 0.023
p = 0.032
p = 0.49
p = 0.024
p = 0.029
p = 0.16
p = 0.93
p = 0.017
p = 0.018
p = 0.27
p = 0.38

Fig. S7: The reproductive properties of the two *Globodera pallida* selection populations on six potato varieties. (**a**) The propagation (Pf/Pi) per generation split out for AMPOP02 and AMPOP10. The dashed vertical line separates the three directly descendant generations from the other two generations. The significances are calculated by a Kruskal-Wallis Rank Sum Test. If significant, there are differences between generations. (**b**) As in (**a**) but for the relative susceptibility. (**c**) as in (**a**) but for the number of eggs per cyst. Each box represents the interquartile range (Q1–Q3; IQR), the horizontal line inside the box marks the median, and the whiskers extend to the smallest and largest non-outlier values (within 1.5× the IQR from the quartiles).

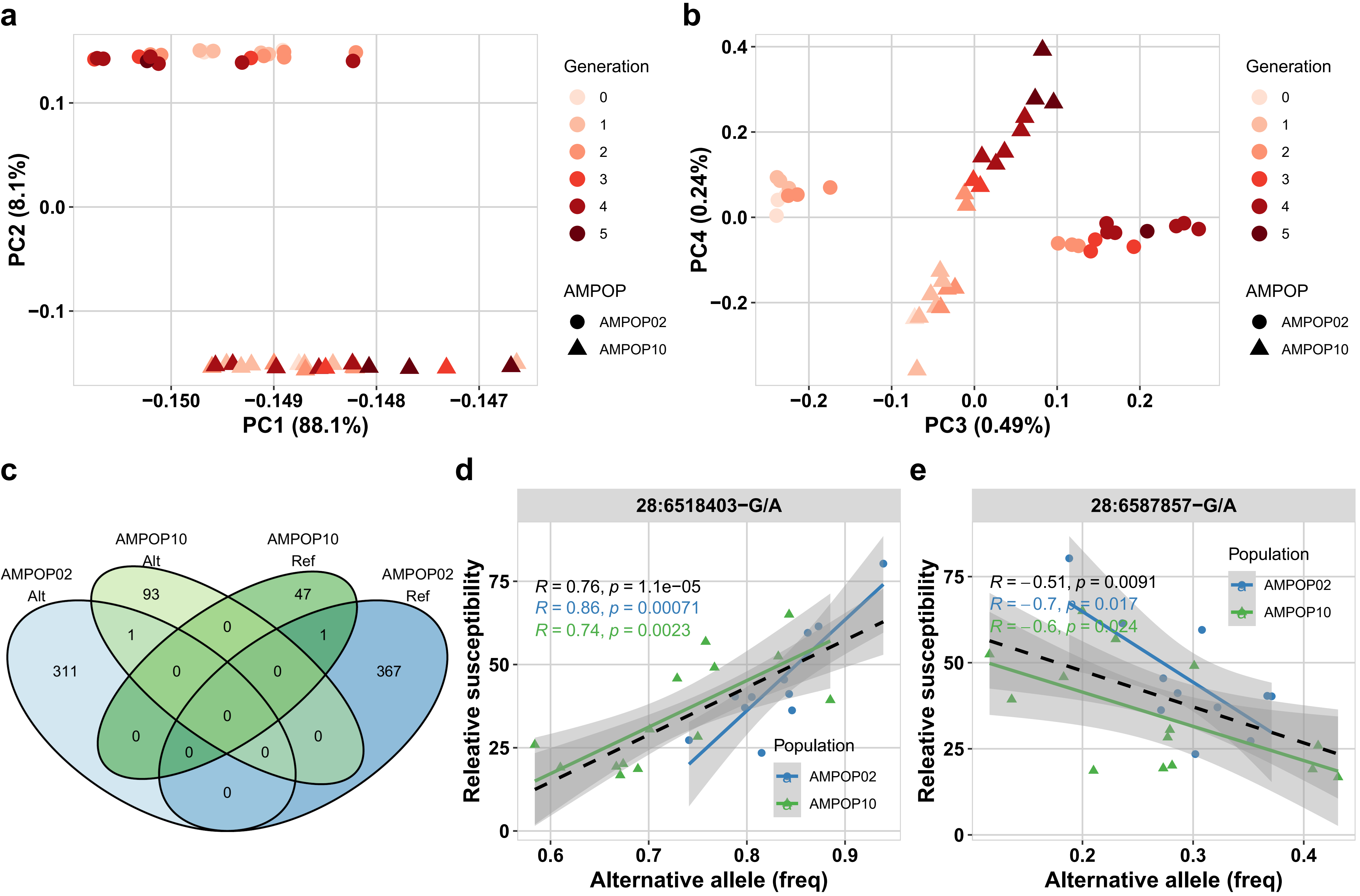
a
b
c
d
e
PC1 (88.1%)
PC2 (8.1%)
−0.150
−0.149
−0.148
−0.147
0.1
0.0
−0.1
PC3 (0.49%)
PC4 (0.24%)
0.4
0.2
0.0
−0.2
−0.2
−0.1
0.0
0.1
0.2
Generation
0
1
2
3
4
5
AMPOP
AMPOP02
AMPOP10
AMPOP02
Alt
AMPOP10
Alt
AMPOP10
Ref
AMPOP02
Ref
311
93
47
367
1
0
0
1
0
0
0
0
0
0
0
0
28:6518403−G/A
R = 0.76, p = 1.1e−05
R = 0.86, p = 0.00071
R = 0.74, p = 0.0023
Population
AMPOP02
AMPOP10
Releative susceptibility
Alternative allele (freq)
0.6
0.7
0.8
0.9
0
25
50
75
28:6587857−G/A
R = −0.51, p = 0.0091
R = −0.7, p = 0.017
R = −0.6, p = 0.024
0.2
0.3
0.4

Fig. S8: Identification of the Seresta-selected loci in *Globodera pallida* populations AMPOP02 and AMPOP10 based on the *G. pallida* Rookmaker genome. (**a**) Principal component analysis on the allele frequencies in the sequenced samples of the selection experiment. The first and second principal components (PC) were associated with population used for the selection and together captured 96.2% of variance in the data. Colours indicate generations, samples from AMPOP02 are indicated with a triangle, samples from AMPOP10 with a circle. (**b**) Principal component analysis on the allele frequencies in the sequenced samples. The third and fourth principal components (PC) were associated with generation of selection and together captured 0.73% of variance in the data. Colours indicate generations, samples from AMPOP02 are indicated with a triangle, samples from AMPOP10 with a circle. (**c**) The overlap in significant variants from the linear model over the generations in AMPOP02 and AMPOP10, split out for increases in Alternative and Reference alleles. Variants derived from AMPOP02 (680) are coloured blue, variants derived from AMPOP10 (142) are coloured green. (**d**) The association of allele frequency of the G nucleotide at position 6518403 of scaffold 28 with relative susceptibility as measured in the fourth resistance pot test. Each dot represents a sample of AMPOP02 and each triangle a sample of AMPOP10. The overall correlation is shown (black dashed line) as well as the correlation of the AMPOP02 (green solid line) and AMPOP10 (blue solid line). Also, the correlation coefficients and significances are given. (**e**) As in (**d**) but for the allele frequency of the A nucleotide at position 6587857 of scaffold 28.

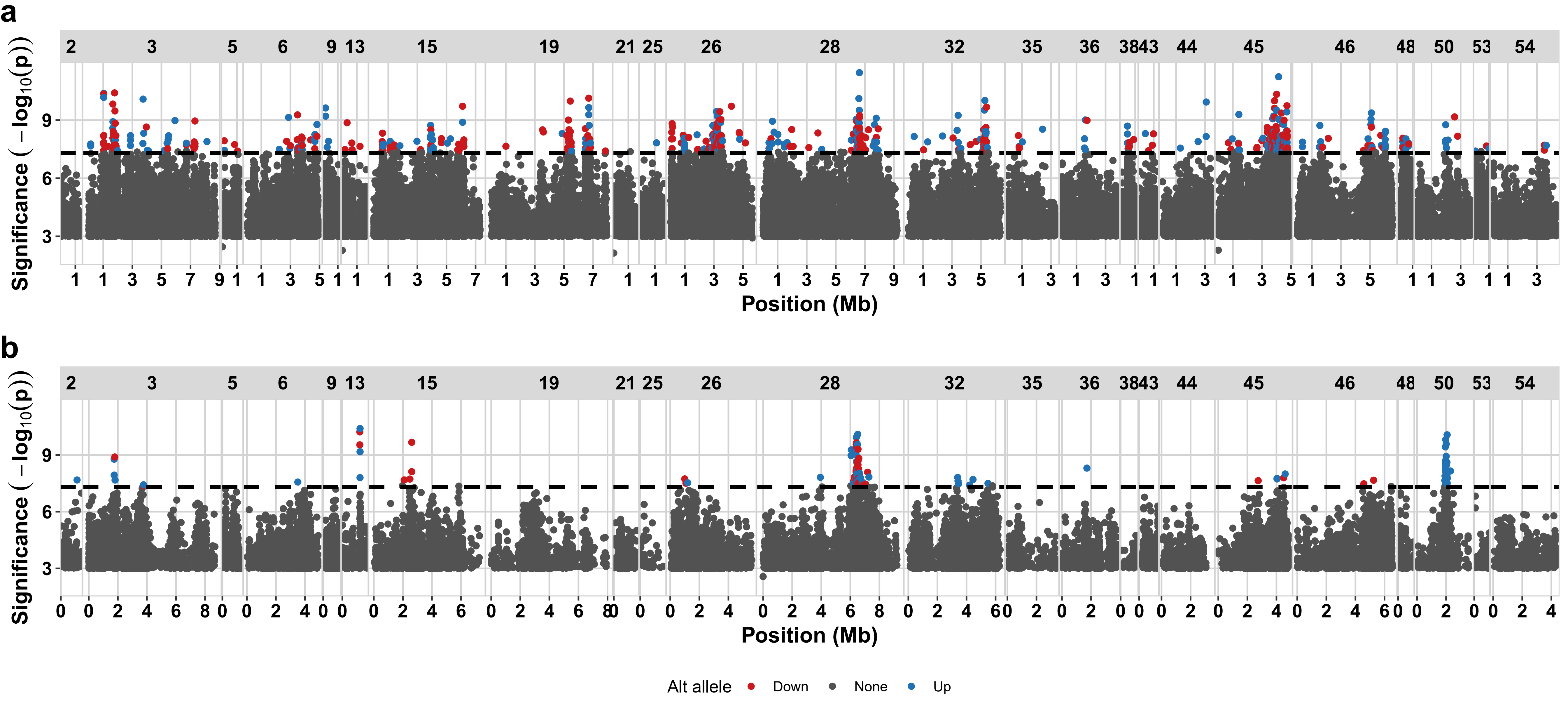
a
b
2 3 5 6 9 13 15 19 21 25 26 28 32 35 36 38 43 44 45 46 48 50 53 54
Significance (−log10(p))
Position (Mb)
Alt allele
Down
None
Up

Fig. S9: Analysis for variants on the *Globodera pallida* Rookmaker genome associated with generation. (**a**) A Manhattan plot of all significances associated with generation in AMPOP02. The variants are plotted on their location (in Mb) versus the significance in $-\log_{10}(p)$. The variants where the frequency of the alternative allele is decreasing over generations are coloured red, where the alternative allele is increasing are coloured blue. Grey variants are not significant. Note that the y-axis has been cut off at $-\log_{10}(p) < 3$ and only scaffolds larger than 1 Mb are shown. (**b**) As in (**a**), but for the variants associated with generation in AMPOP10.

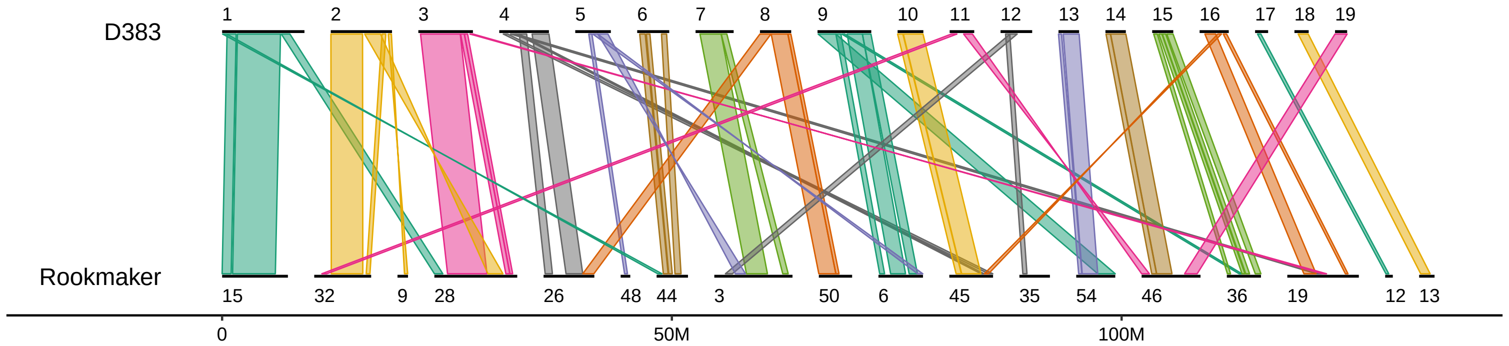
D383
1
2
3
4
5
6
7
8
9
10
11
12
13
14
15
16
17
18
19
Rookmaker
15
32
9
28
26
48
44
3
50
6
45
35
54
46
36
19
12
13
0
50M
100M

Fig. S10: Synteny plot between the *Globodera pallida* D383 genome and the *G. pallida* Rookmaker genome. Only syntenic blocks larger than 300 kb of D383 scaffolds 1-19 were visualised.

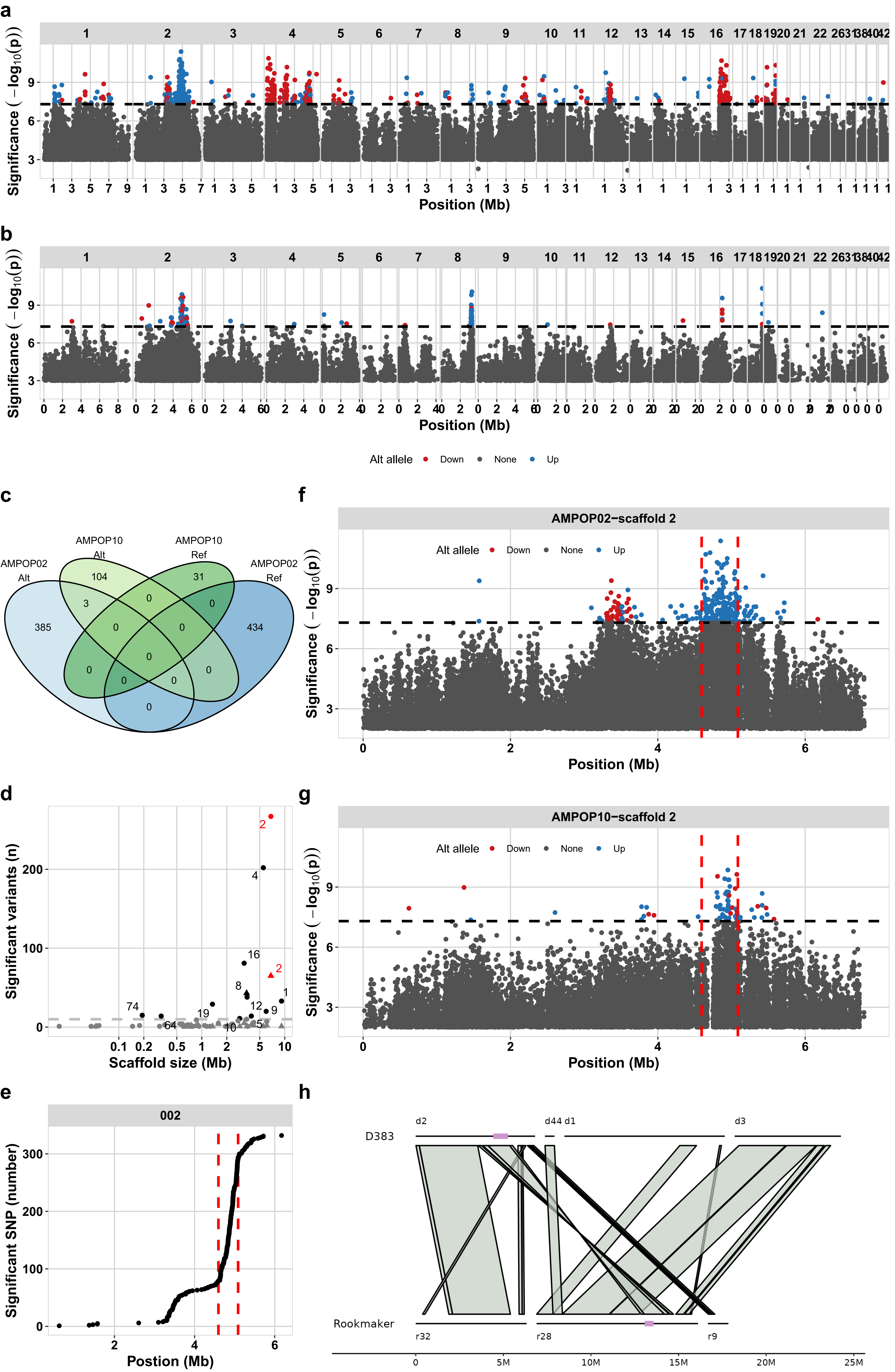
a
b
Significance (−log10(p))
Position (Mb)
Alt allele Down None Up
c
AMPOP02 Alt
AMPOP10 Alt
AMPOP10 Ref
AMPOP02 Ref
385
104
31
434
3
d
Significant variants (n)
Scaffold size (Mb)
e
002
Significant SNP (number)
Postion (Mb)
f
AMPOP02−scaffold 2
g
AMPOP10−scaffold 2
h
D383
d2
d44 d1
d3
Rookmaker
r32
r28
r9
0
5M
10M
15M
20M
25M

Fig. S11: A region associated with virulence on *GpaV*$_{vrn}$, syntenic to *Globodera pallida* Rookmaker Scaffold 28 identified on the D383 genome. (**a**) A Manhattan plot of all significances associated with generation in AMPOP02. The variants are plotted on their location (in Mb) versus the significance in $-\log_{10}(p)$. The variants where the frequency of the alternative allele is decreasing over generations are coloured red, where the alternative allele is increasing are coloured blue. Grey variants are not significant. Note that the y-axis has been cut off at $-\log_{10}(p) < 3$ and only scaffolds larger than 1 Mb are shown. (**b**) As in (**a**), but for the variants associated with generation in AMPOP10. (**c**) The overlap in significant variants from the linear model over the generations in AMPOP02 and AMPOP10, split out for increases in Alternative and Reference alleles. Variants derived from AMPOP02 (822) are coloured blue, variants derived from AMPOP10 (138) are coloured green. (**d**) The number of variants identified per scaffold, with the scaffold-size on the x-axis (in million bases; Mb) and the number of significant variants on the y-axis. The horizontal dashed grey line indicates 10 variants per scaffold. Circles indicate the variants were found in the AMPOP02 population and triangles indicate variants were found in the AMPOP10 population. Text indicates which scaffold of the D383 genome the datapoint belongs to. (**e**) Identification of the locus most likely to harbour the causal virulence gene(s) based on the significantly associated variants in AMPOP02 and AMPOP10. The x-axis indicates the physical position in million bases (Mb) and the y-axis the order-number (1 - 325). The dashed vertical lines indicate the candidate locus as determined by changepoint-analysis. (**f**) The significance of the variants on scaffold 2 associated with generation in AMPOP02. The variants are plotted on their location on scaffold 2 (in Mb) versus the significance in $-\log_{10}(p)$. The variants where the frequency of the alternative allele is decreasing over generations are coloured red, where the alternative allele is increasing are coloured blue. Grey variants are not significant. Note that the y-axis has been cut off at $-\log_{10}(p) < 2$. (**g**) As in (**f**), but for the variants associated with generation in AMPOP10. (**h**) A synteny plot of scaffold 2 of the D383 genome and scaffold 28 of the Rookmaker genome, with the virulence loci indicated by pink boxes. The annotations indicate which scaffolds are shown (e.g. d2 is scaffold 2 of the D383 genome). Although the genomic context of the two virulence loci differs between the two genomes, both association analyses landed on syntenic regions of the genome. The x-axis indicates the scale in million bases (M).

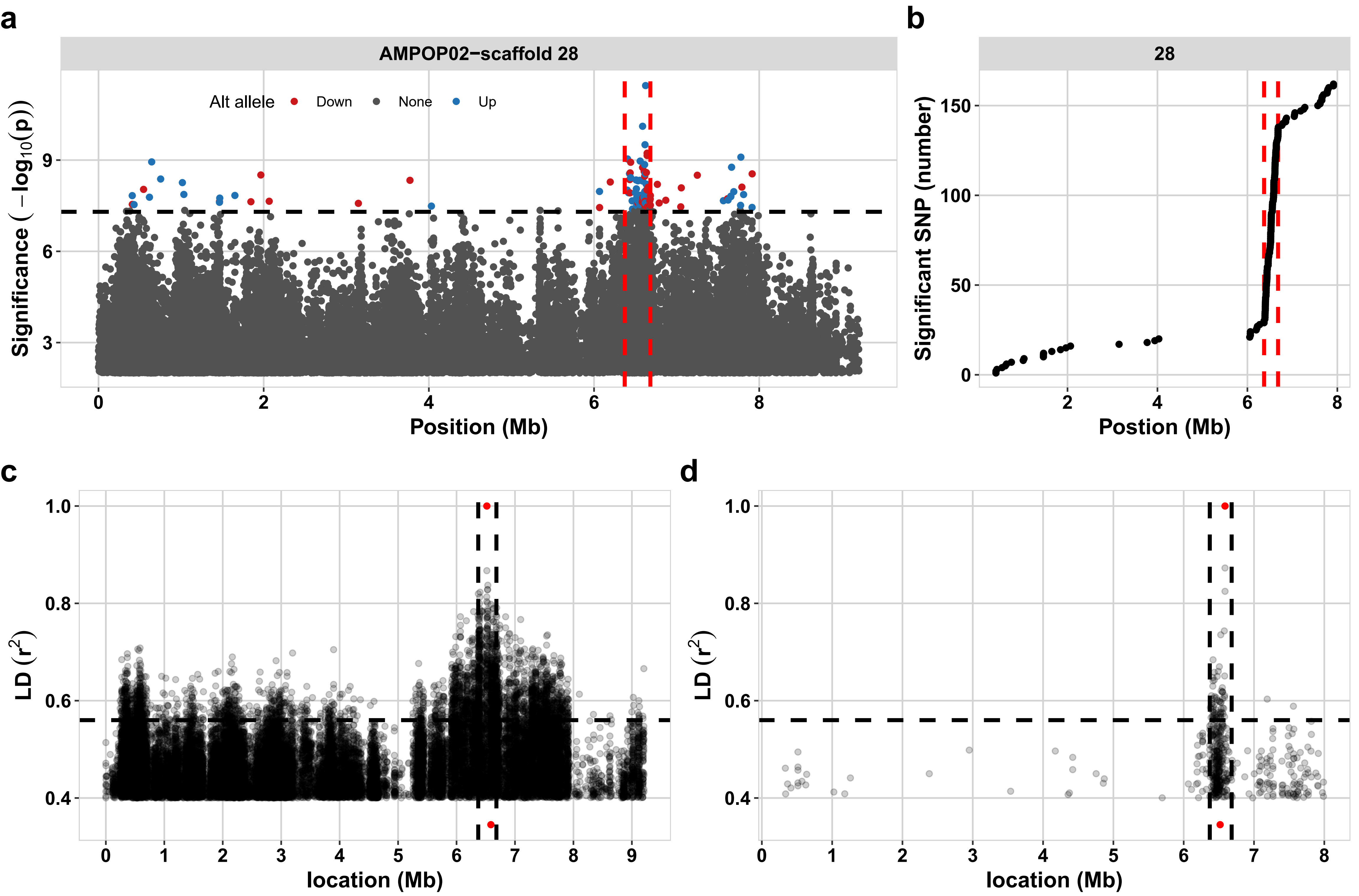

a
AMPOP02−scaffold 28
Alt allele
Down
None
Up
Significance (−log10(p))
Position (Mb)
b
28
Significant SNP (number)
Postion (Mb)
c
LD (r2)
location (Mb)
d
LD (r2)
location (Mb)

Fig. S12: Region on scaffold 28 of the *Globodera pallida* Rookmaker genome associated with virulence. (**a**) The significance of the variants on scaffold 28 associated with generation in AMPOP02. The variants are plotted on their location on scaffold 28 (in Mb) versus the significance in $-\log_{10}(p)$. The variants where the frequency of the alternative allele is decreasing over generations are coloured red, where the alternative allele is increasing are coloured blue. Grey variants are not significant. The dashed horizontal line indicates the Bonferroni-corrected threshold. The dashed vertical lines indicate the candidate locus. Note that the y-axis has been cut off at $-\log_{10}(p) < 2$. (**b**) Identification of the locus most likely to harbour the causal virulence gene(s) based on the significantly associated variants in AMPOP02 and AMPOP10. The x-axis indicates the physical position in million bases (Mb) and the y-axis the order-number (1 - 162). The dashed vertical lines indicate the candidate locus as determined by changepoint-analysis. (**c**) Linkage between the G nucleotide at position 6518403 of scaffold 28 and the variants on scaffold 28. The x-axis shows the position of the variants in million bases (Mb). The y-axis shows the squared Pearson correlation. The y-axis was cut-off at $R^2 < 0.4$. The dashed horizontal line indicates the threshold of the 1% best-correlating variants ($R^2 = 0.56$). The dashed vertical lines indicate the area where the virulence gene is likely to be located as determined by changepoint analysis. The two virulence-associated variants are coloured red. Note that the two have a low correlation ($R^2 = 0.35$). (**d**) As in (**c**) but for the A nucleotide at position 6587857 of scaffold 28.

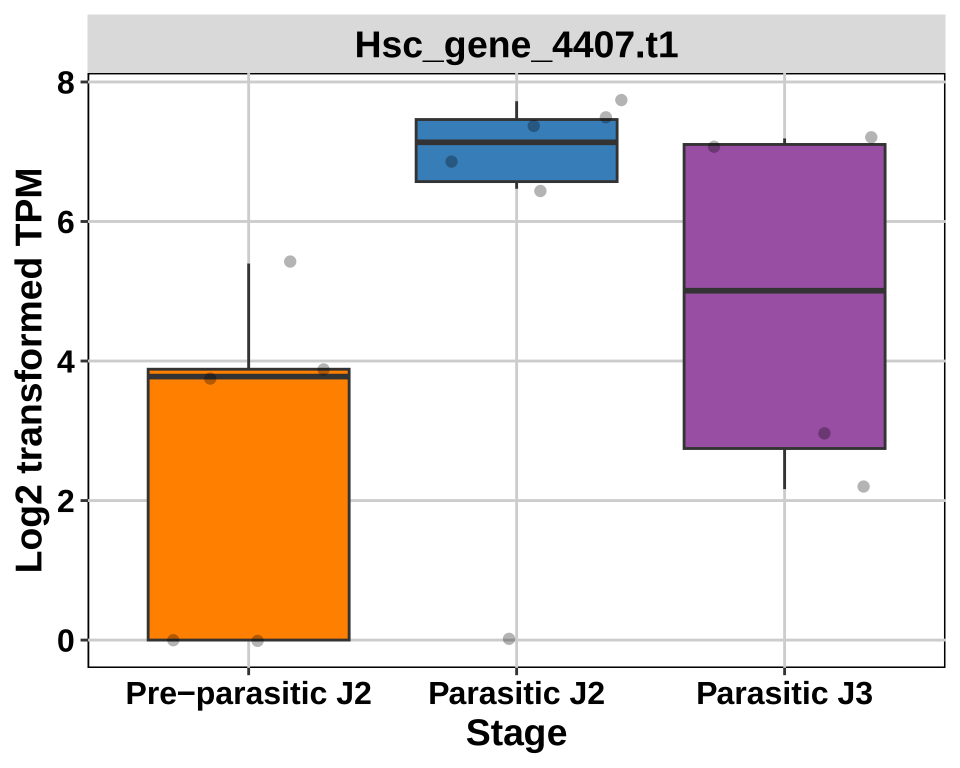

Hsc_gene_4407.t1
Log2 transformed TPM
0
2
4
6
8
Pre−parasitic J2
Parasitic J2
Parasitic J3
Stage

Fig. S13: Gland cell expression of the *Heterodera schachtii* gene *Hsc_gene_g4407* across three distinct stages. This putative poly-A polymerase is the best BLASTp hit of *Gp-pat-1* on the *H. schachtii* genome. Data obtained from Molloy et al. (2024). Each box represents the interquartile range (Q1–Q3; IQR), the horizontal line inside the box marks the median, and the whiskers extend to the smallest and largest non-outlier values (within 1.5× the IQR from the quartiles).

SUPPLEMENTARY NOTES

Note S1

Manual genome annotation of the avirulence locus

Within the 311 kb region of interest on the Rookmaker genome, automated genome annotation predicted 53 transcripts on 48 genes. Because of the inherent limitations of automated genome annotations, the region of interest was manually inspected and curated as described by Moya et al. (2023).

In the manual curation process, 19 transcripts maintained their Braker predictions, and an additional 23 transcripts had only untranslated regions (UTRs) added. For 5 transcripts, structural changes were applied to one or more exons. Furthermore, 3 transcripts resulted from gene fusion events and 4 transcripts emerged from the splitting of two genes. Notably, 19 extra transcripts were predicted for genes initially identified by Braker, and the manual curation process identified 3 genes not identified by Braker. This curation effort yielded a total of 76 transcripts for 47 genes.

References

Molloy, B., Shin, D. S., Long, J., Pellegrin, C., Senatori, B., Vieira, P., Thorpe, P. J., Damm, A., Ahmad, M., Vermeulen, K., Derevnina, L., Wei, S., Sperling, A., Reyes Estévez, E., Bruty, S., de Souza, V. H. M., Kranse, O. P., Maier, T., Baum, T., & Eves-van den Akker, S. (2024). The origin, deployment, and evolution of a plant-parasitic nematode effectorome. *PLOS Pathogens*, *20*(7), e1012395. https://doi.org/10.1371/journal.ppat.1012395

Moya, N. D., Stevens, L., Miller, I. R., Sokol, C. E., Galindo, J. L., Bardas, A. D., Koh, E. S. H., Rozenich, J., Yeo, C., Xu, M., & Andersen, E. C. (2023). Novel and improved Caenorhabditis briggsae gene models generated by community curation. *BMC Genomics*, *24*(1), 486. https://doi.org/10.1186/s12864-023-09582-0
